# Suppression of apoptosis impairs phalangeal joint formation in the pathogenesis of brachydactyly type A1

**DOI:** 10.1038/s41467-024-45053-0

**Published:** 2024-03-12

**Authors:** Adrian On Wah Leung, Andrew Chung Hin Poon, Xue Wang, Chen Feng, Peikai Chen, Zhengfan Zheng, Michael KaiTsun To, Wilson Cheuk Wing Chan, Martin Cheung, Danny Chan

**Affiliations:** 1 School of Biomedical Sciences, The University of Hong Kong, Pokfulam Hong Kong, China; 2 Hebei Orthopedic Clinical Research Center, The Third Hospital of Hebei Medical University, 050051 Shijiazhuang, Hebei China; 3 Department of Orthopaedics Surgery and Traumatology, The University of Hong Kong -Shenzhen Hospital (HKU-SZH), Shenzhen, China; 4 Department of Orthopaedics and Traumatology, The University of Hong Kong, Pokfulam Hong Kong, China

**Keywords:** Cartilage development, Limb development, Body patterning

## Abstract

Apoptosis occurs during development when a separation of tissues is needed. Synovial joint formation is initiated at the presumptive site (interzone) within a cartilage anlagen, with changes in cellular differentiation leading to cavitation and tissue separation. Apoptosis has been detected in phalangeal joints during development, but its role and regulation have not been defined. Here, we use a mouse model of brachydactyly type A1 (BDA1) with an Ihh^E95K^ mutation, to show that a missing middle phalangeal bone is due to the failure of the developing joint to cavitate, associated with reduced apoptosis, and a joint is not formed. We showed an intricate relationship between IHH and interacting partners, CDON and GAS1, in the interzone that regulates apoptosis. We propose a model in which CDON/GAS1 may act as dependence receptors in this context. Normally, the IHH level is low at the center of the interzone, enabling the “ligand-free” CDON/GAS1 to activate cell death for cavitation. In BDA1, a high concentration of IHH suppresses apoptosis. Our findings provided new insights into the role of IHH and CDON in joint formation, with relevance to hedgehog signaling in developmental biology and diseases.

## Abstract (web summary)

Authors explore the biological mechanisms underlying a missing digit joint in Brachydactyly type A1. They showed that excess Indian Hedgehog proteins suppress the apoptosis level needed for the progression of digit joint development in a mouse model.

## Introduction

Limb bones are formed sequentially from proximal to distal in development, and different bones are separated through the formation of synovial joints^1^. While the cellular events are known, the molecular regulation of joint (interzone) initiation and progression is less clear^2^. Progression is a balance between chondrogenic (BMP/TGFβ^3,4^) and non-chondrogenic (WNT^5–8^) signals. FGF and IHH signaling are also involved^9–12^. *Wnt9a* and *Gdf5* are markers of early interzone cells, lubricin (*Prg4*), *CD44*, and *Col2a1* of a later stage, and *Lgr5* and *Col22a1* of intermediate to late stages of joint formation prior to cavitation^3,13–16^.

Cavitation is the physical separation of the cartilage templates, and failure leads to joint fusions. Genetic disorders of joint formation include the brachydactylies, with short digits and missing or fused joints^17^. Factors involved include the TGFβ superfamily, both receptors and antagonists as well as SHH signaling^18–20^. *IHH* is associated with brachydactyly type A1 (BDA1)^21–24^. The relationship of type A1 to other forms of brachydactyly is not clear, but upstream to BMP signaling is implicated^17,25^.

Studies of BDA1 mutations^21,26^ have shown an impaired interaction of mutant IHH protein with the receptor, Patched 1 (PTCH1), and the negative regulator, hedgehog interacting protein (HHIP), reducing IHH signaling capacity but increasing its signaling range^21,27,28^. *Hhip* expressed at the boundaries of the interzone restricts the amount of IHH that can enter. In *Ihh*^*E95K*^ BDA1 mice, IHH travels further into the developing interzone region, with an overall increase in canonical IHH signaling, resulting in a higher level of PTHrP that signals to the distal digit tip, impairing growth of the phalangeal cartilage templates and producing the shortened middle phalangeal bone pathology in digits II, III, and IV^21^. However, the cause of the missing joint in the forelimb digit V is unresolved. It might be that the length of the distal cartilage template is too short for a joint to form, as seen in chick and mouse digit development^29–32^. Another possibility is a failure of joint cavitation.

Cavitation is an important process in joint development, but the mechanism is not well understood. Apoptosis has been proposed to occur within the interzone^33–35^, and observed in the developing phalangeal joints in mice^36–38^. However, apoptosis was not detected in the developing knee or elbow joints in rats and rabbits^39,40^. The requirement for cavitation could differ between knee/elbow and phalangeal joints, and this would be consistent with the fact that in BDA1 patients, only phalangeal joints are affected.

IHH in skeletal biology is best known for regulating chondrocyte proliferation and hypertrophy in endochondral ossification^41,42^. In the canonical pathway, IHH signals via Smoothened (SMO) to stabilize the Gli transcriptional complex in activating downstream targets^43,44^. IHH also interacts with Cell adhesion molecule-related/downregulated by oncogenes (CDON), Biregional Cdon-binding protein or brother of Cdon (BOC), and Growth arrest specific-1 (GAS1)^45^. Together, these establish a gradient of hedgehogs with positional values in development. Thus, understanding the impact of IHH mutations on the interaction with these partners will provide insights into the biology and pathogenesis of BDA1.

CDON, BOC, and GAS1 form distinct complexes with PTCH1 and are required for hedgehog (Hh)-mediated cellular functions^45,46^. PTCH1^47,48^ and CDON^49–51^ have characteristics of dependence receptors which are proteins that mediate programmed cell death by monitoring the absence of certain trophic factors. Thus, in vitro, PTCH1 can trigger an apoptotic event in the absence of Hh through the recruitment of a caspase-activating complex, via its intracellular domain^52^. Similarly, ligand-free CDON can trigger apoptosis in vitro via activation of caspase-9^49^. GAS1 is a GPI-anchored membrane protein with no intracellular domains that has been shown to extend the range of Hh signaling by facilitation^53^. GAS1 can induce cell death via the intrinsic apoptosis pathway, triggering dephosphorylation of BAD with no activation of caspase-8^54,55^. Thus, GAS1 can function as a dependence receptor, even in the absence of an intracellular domain.

Here, we investigated the impact of BDA1 IHH mutations on the interaction with its partners. We showed that the interactions with PTCH1 and HHIP are impaired to a similar extent and those with CDON and GAS1 to a lesser degree. We confirmed that apoptosis contributes to cavitation during the formation of phalangeal joints, in a process that involves CDON functioning as a potential dependence receptor when the IHH level is low. In the BDA1 mouse, we showed phalangeal joints in forelimb digit V are initiated, but did not progress due to impaired cavitation, linked to reduced apoptosis, and a joint is lost. These findings have broad relevance, as hedgehog signaling is involved in many developmental processes and diseases, including cancer progression and metastasis.

## Results

### IHH mutations cause BDA1 to share common defects for interaction with partner molecules

Mutations in IHH-causing BDA1 to affect both signaling capacity and range due to impaired interactions with its receptor, PTCH1, and its negative regulator for the range of diffusion, HHIP^21,27,28^. The model for the molecular and cellular outcomes leading to a missing joint in BDA1 was based on the impairment of interactions with these two partners of opposing effects^21^. However, contributions from impaired interaction with potentiators of Hh signaling, such as GAS1, CDON, and BOC, have not been studied, and the commonality between the different IHH mutations is not well understood. Previously, we have shown the mRNA expression of *Ihh*, *Ptch1*, and *Hhip* in the developing mouse digits^21^. To gain a more complete picture of IHH signaling, we performed immunostaining for GAS1 and CDON and showed that they are expressed in the developing interzones at embryonic day 14.5 (E14.5) (Supplementary Fig. S1). Thus, the model for the pathology of BDA1 and related brachydactyly disorders needs to include an assessment of these partners (Supplementary Fig. S2).

Here, we evaluate four IHH missense mutations causing BDA1 (E95K, D100E, R128Q, and T154I)^23,24,26,56^, and a missense mutation, P46L, causing Acrocapitofemoral Dysplasia (ACFD)^57^. BDA1 is an autosomal dominant disorder with varying combinations of shortened middle phalanges and/or missing digit joints;^21,58^ whereas ACFD is an autosomal recessive disorder with cone-shaped epiphyses in hands and hips, with shortened middle phalange, but no missing joints^57,59^.

**Fig. 1:**
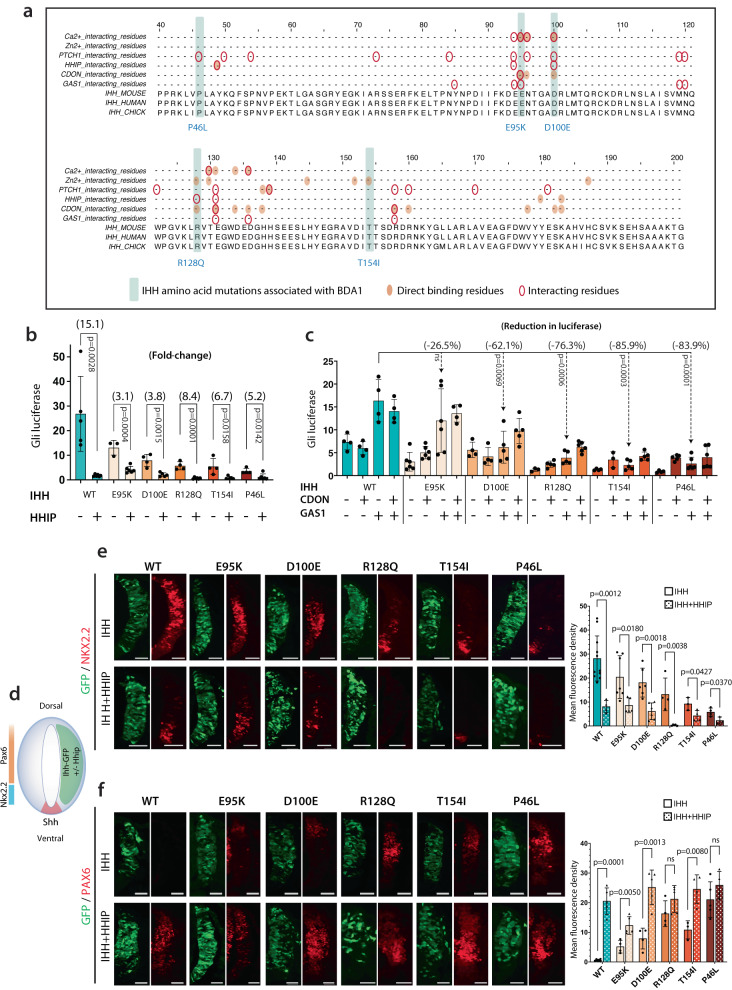
General reduction of IHH signaling and HHIP inhibition in BDA1 mutations with elongated Group I signaling field. **a** Multiple alignment of mouse, human, and chicken IHH protein sequences. The location of five amino acid mutations associated with BDA1, binding, and interacting residues based on information from literature are highlighted. **b** In the presence of HHIP, the IHH signal was generally downregulated in both WT and mutant IHH according to the Gli-luciferase assay. The inhibitory effect of HHIP on mutant Hh was not comparable to that on WT Hh. **c** Gli1-firefly luciferase assay of BDA1 mutations with CDON and GAS1 overexpression, demonstrating reduced interactions between mutant IHH and GAS1. CDON showed no effect alone or in concert with GAS1 on the signaling activities of WT and mutant IHHs. **d** WT and BDA1 IHH constructs were electroporated with/without HHIP to one side of the chick neural tube (HH 11-12), and the known expression regions of NKX2.2 and PAX6 in the control half are illustrated. **e** Overexpression of WT IHH induced expression of ventral marker NKX2.2 in the dorsal region, this effect was weaker in all IHH mutants. NKX2.2 expression was restricted to the ventral region when WT-IHH and HHIP were co-expressed. A similar pattern was observed in E95K and D100E mutations. NKX2.2 expression in the remaining three IHH mutations was abolished in the presence of HHIP. Scale bar = 100 μm. Mean fluorescence densities from experimental replicates are shown in the bar chart. **f** Overexpression of WT IHH suppressed expression of dorsal marker PAX6. Weaker suppression of Pax6 in mutant IHHs was observed. Co-expression of IHH and HHIP restored PAX6 expression in both WT and mutant IHH. Scale bar = 100 μm. For the bar chart in **b**, **c**, **d**, and **f**, each data point represents an independent biological replicate, bar height indicates mean and error bars indicate standard deviations. *p*-values are calculated with a two-sided student’s *t*-test. Source data are provided as a Source Data file.

Mapping these five mutations onto the crystal structure of SHH^24,28,57^, which has a protein sequence highly homologous to IHH, showed potential disruption of interacting sites with partners, or of a pseudo-active site arising from coordination of two Ca^2+^ ions and one Zn^2+^ ion in their respective interacting grooves (Supplementary Fig. S3)^46,60,61^. The Ca^2+^ groove is necessary for interaction with all Hh partners, and metal ion chelation significantly inhibits Hh interaction with CDON and BOC^60,62^. The positions of the mutations relative to the proposed functional residues at the interaction site are visualized in a linear sequence comparison (Fig. 1a). E95K, D100E, and R128Q are located within the main cluster of interaction sites, while T154I is located at coordination sites for the Zn^2+^ ion, and P46L is near the PTCH1 and HHIP interaction sites (Fig. 1a).

Next, we used the Gli-binding sites (GBS)-driven luciferase reporter construct (GBS-Luc) to assess the impact of different IHH mutations and their association with HHIP on Hh signaling activity in the developing chick neural tube (Fig. 1b). Wild-type (WT) IHH induced robust luciferase activity, whereas HHIP suppressed IHH signaling activity by 15-fold. In contrast, E95K, D100E, R128Q, T154I, and P46L showed a “graded” reduction of their efficacy in activating luciferase expression, and inclusion of HHIP further dampened their signaling activities with a 3.1- to 8.4-fold reduction (Fig. 1b). HHIP inhibited IHH signaling to a similar degree regardless of the location of the IHH mutation. GAS1 potentiated WT IHH signaling but this effect was reduced in the IHH mutants (from 26.5% to 83.9%) compared to their effects on WT IHH (Fig. 1c). CDON showed no effect alone or in concert with GAS1 on the signaling activities of WT and mutant IHHs (Fig. 1c). These results suggest that GAS1 promotes IHH signaling, and this is diminished by the BDA1 and ACFD mutations (Fig. 1c).

### BDA1 mutant IHH signaling capacities are suppressed by HHIP irrespective of mutation sites

To assess the signaling capacity of different IHH mutations in the chick neural tube, we performed in ovo electroporation to examine their ability to induce expression of a Class II transcription factor, NKX2.2, activated by SHH signaling in the ventral region of the neural tube, and to repress expression of a Class I transcription factor, PAX6, which is inhibited by high levels of SHH in the intermediate region of the neural tube^63^ (Fig. 1d).

Overexpression of WT *Ihh* induced ectopic NKX2.2 expression along the dorsal-ventral axis (Fig. 1e). Co-expression of HHIP significantly suppressed the induction of NKX2.2 in the dorsal region, consistent with previous observations^21^. Overexpression of IHH mutants resulted in a graded reduction in ectopic NKX2.2 expression ranging from moderate (E95K, D100E, R128Q) to weaker effects (T154I and P46L). Co-expression of HHIP inhibited the dorsal expansion of NKX2.2 expression by E95K and D100E to a similar extent as WT IHH and completely abolished the ability of R128Q, T154I, and P46L to induce ectopic NKX2.2 expression (Fig. 1e). These results are in line with the luciferase assays (Fig. 1b).

In contrast, opposite responses to the ectopic expression of IHH mutants with or without HHIP were observed for PAX6 expression. HHIP inhibited the ability of ectopic WT IHH to completely abolish PAX6 expression in the intermediate region of the neural tube. E95K and D100E also completely inhibited PAX6 expression but R128Q, T154I, and P46L showed moderate inhibition. Co-electroporation of HHIP and IHH mutants restored most of the endogenous PAX6 expression (Fig. 1f). Altogether, these results suggest that distinct IHH mutants reduce signaling capacity to different extents, which can be further diminished by HHIP regardless of the mutation sites.

### Missing distal phalangeal joint in BDA1 is due to failure of cavitation in development

To investigate the basis of missing phalangeal joints in BDA1 patients, we used our *Ihh*^*E95K*^ BDA1 mouse model^21^. Mice homozygous for the *E95K* allele exhibited typical BDA1 characteristics (referred to as BDA1 mice hereon), with shortened middle phalangeal bones in digits II, III, and IV, but a missing phalangeal joint in digit V^21,22^. We investigated digit III as the reference with a shortened middle phalangeal from E15.5 to newborn and showed that all phalangeal joints (M/P1, P1/P2, and P2/P3) were initiated and progressed to cavitation, with all joint tissues formed at birth; albeit about one day later than in WT mice (Supplementary Fig. S4a, b).

**Fig. 2:**
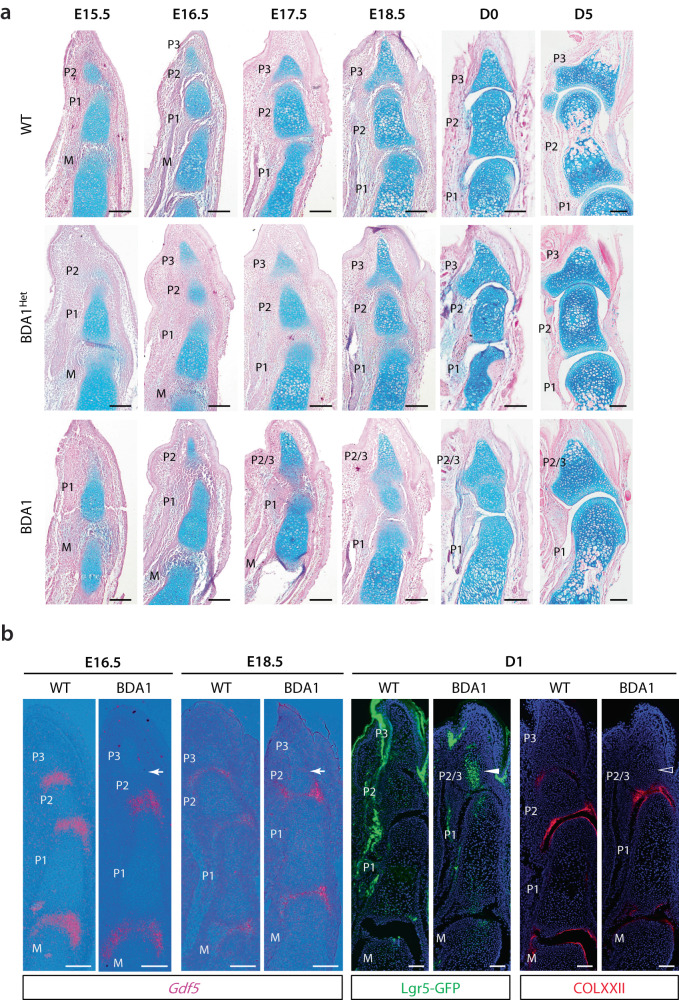
Formation of distal BDA1 joint is initiated but failed to cavitate. **a** Representative histological images (stained with Alcian blue and nuclear fast red) of WT, BDA1^Het^, and BDA1 digit V from embryonic days E15.5 to postnatal Day 5 (D5), (*n* = 3). Initiation of interzone formation but lack of distal joint cavitation was observed in BDA1 mice. Scale bar = 100 µm. **b** Expression of interzone marker *Gdf5* (in situ hybridization) was delayed or decreased (arrows) in the distal joint of BDA1 digit V (*n* = 3). *Lgr5*-expressing cells were detected in the presumptive P2/P3 interzone (solid arrow) (*n* = 3), and no articular surface marker Collagen XXII was detected (open arrow) (*n* = 3) in postnatal Day1 (D1) P2/3 joint of BDA1. Scale bar = 100 µm.

In mice heterozygous for the *E95K* allele (BDA1^het^), all phalangeal joints are formed in digit V but the middle phalangeal bone is shorter than WT, the developmental process is delayed (Fig. 2a). In BDA1 mice, the formation and progression of M/P1 and P1/P2 joint developed similarly, with clear separation of the Alcian blue stained cartilage anlagen by E15.5, and cavitation at E18.5 (Fig. 2a). Formation of the P2/P3 interzone in WT digit V occurs at E15.5 and at E16.5 for BDA1^het^ mice. In the BDA1 mice, there was histological evidence of early flattened interzone cells and reduced Alcian blue staining at the presumptive future P2/P3 site at E18.5 (Fig. 2a). Thus, a P2/P3 interzone is initiated in BDA1 mice but with a marked developmental delay.

At birth (D0), all phalangeal interzones in WT and BDA1^het^ mice have cavitated and the Alcian blue-positive cartilage anlagen are well separated, with a distinct joint space (Fig. 2a). In BDA1 mice, the P2/P3 interzone remained condensed at the presumptive site, and cells had reverted to a more rounded chondrogenic phenotype with no evidence of cavitation (Fig. 2a). By postnatal day 5 (D5), the BDA1 mice showed no evidence of the P2/P3 interzone, presumably fused, but the more proximal P1/P2 joint had developed (Fig. 2a). The P2/P3 joint was missing in digit V for all 20 postnatal BDA1 mice investigated by histological and radiography. The number of phalangeal bones does not change in adult life (Supplementary Fig. S4c).

This finding is contrary to the hypothesis that the P2/P3 joint cannot be initiated due to impaired distal growth and shortening of the cartilage anlagen. While we showed that failure to initiate a joint can be a mechanism in a brachydactyly type B1 (BDB1) mouse model carrying a *Ror2*^*W749X*^ mutant allele^22,64^ (Supplementary Fig. S5), this is not the case in the BDA1 mouse. Instead, the data points to altered cellular activities within the developing P2/P3 interzone in BDA1 mice.

Next, we hypothesized that excessive Hh signaling in the interzone leads to impaired joint formation and investigated the joint-forming process in the *short digits* (*Dsh*) mouse^18^. *Dsh* is a radiation-induced mutant with an inversion in the *Shh* locus that causes ectopic expression of *Shh* during digit development, at the time of the P1/P2 interzone formation^18^. When we investigated the formation of the P1/P2 interzone, we saw the initiation and early progression of this joint to E17.5, but no cavitation, and the interzone progression ceased by E18.5, with cells reverting to chondrocyte-like, and the joint was missing (Supplementary Fig. S5). The P2/P3 interzone was initiated at E16.5, expanded, and progressed to cavitation by E18.5, and this joint was formed. Thus, in *Dsh* mice, the digit phenotype is due to failure to form the P1/P2 joint because of ectopic SHH expression. In BDA1 mice, excessive IHH signaling in the P2/P3 interzone caused abnormal progression that failed to cavitate. In both mouse models, the failure of interzone formation is in part due to excessive Hh signaling in the interzone, altering an event necessary for progression to cavitation, tipping the balance to the chondrogenic lineage, and the joint is not formed. In BDA1 mice, the delayed initiation of the P2/P3 interzone and the failure to progress could be a combination of both the reduced size of the distal cartilage anlagen and abnormal Hh signaling in the interzone.

At the molecular level, *Gdf5* marks the event of interzone formation^14^, with strong expression in the forming phalangeal interzones at E16.5, reducing with maturation and cavitation by E18.5 in WT mice (Fig. 2b). In BDA1 mice, *Gdf5* was detected in the forming M/P1 and P1/P2 interzones with no indication of a P2/P3 interzone as it had not yet initiated (Fig. 2b). Expression decreased by E18.5 in the proximal interzones, with the onset of a weak signal in the presumptive P2/P3 interzone (Fig. 2b), consistent with the histological observation.

*Lgr5* and *Col22a1* mark the later interzone progression prior to cavitation and formation of the articular cartilage layer, respectively^15^. At D1, LGR5 expression has diminished significantly with the expression of Collagen XXII (*Col22a1*) in the cavitating interzone, and is strongly localized to the superficial layer of the juvenile articular cartilage (Fig. 2b). In BDA1, Collagen XXII was not detectable at D1 but a group of *Lgr5*-expressing cells was present in the presumptive P2/P3 interzone. Together, the expression patterns of *Gdf5*, *Lgr5*, and *Col22a1* indicate initiation of the interzone for the P2/P3 joint in BDA1 digit V that fails to progress through cavitation.

### Single-cell transcriptomics identified *Cdon* expression and apoptosis as key molecular and cellular changes

**Fig. 3:**
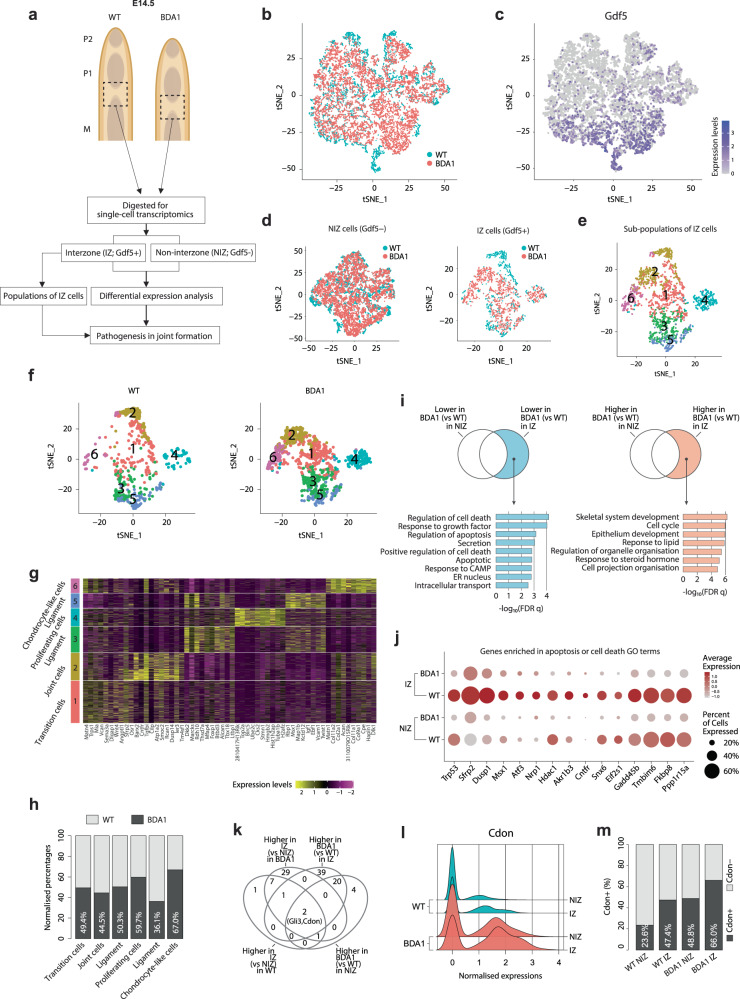
Single-cell transcriptomics identified Cdon and apoptosis as key molecular and cellular changes. **a** Diagram showing the region of digit III in E14.5 WT and BDA1 mice dissected and the workflow for single-cell transcriptomic analyses. **b** A tSNE plot showing the dimension reduction result of the two samples, after canonical correlation analyses were applied to correct the potential batch effects. **c** A tSNE plot showing the normalized expression level of Gdf5 in the two samples. **d** The *Gdf5+* and *Gdf5-* cells were defined as interzone (IZ) and non-interzone (NIZ) cells, respectively; and were separated for training new tSNE results, as shown in the two scatter plots. **e** Six sub-populations of cells were identified in the interzone cells. **f** The cells with origins from the WT (left) and the BDA1 (right) were shown separately. **g** A heatmap showing the top 10 marker genes of each of the six populations in the interzone. **h** Bar chart showing the normalized proportions of cells (as normalized by the total numbers of WT and BDA1 interzone cells) with origins from the BDA1 (dark gray) and WT (light gray), per each of the six populations. Percentages are for the BDA1 cells. **i** Venn diagrams showing identification of genes that are either lower or higher in the BDA1 interzone as compared with the WT interzone cells. Specificity of the genes to the interzone was achieved by filtering genes that were differentially expressed across the two genotypes in the non-interzone cells. The DEGs were further functionally characterized by gene-ontology (GO) analyses, as shown in the representative GO terms. **j** A bubble-plot showing the expression levels and fractions of an aggregate of genes related to the apoptosis or cell death GO terms revealed in **i**). **k** A Venn-diagram showing the DEGs expressed higher in the interzone (as compared with the non-interzone), and/or in the BDA1 (as compared with the WT). Two genes, *Cdon* and *Gli3*, expressed highest in BDA1 interzone. **l** Ridgeplot showing the expression levels of *Cdon* across the four groups of cells, as labeled on the *y*-axis. **m** Bar chart showing the normalized proportions of cells expressing *Cdon* in the four groups of cells as labeled on the *x*-axis.

To investigate the initial events leading to failure of P2/P3 joint formation in digit V, we performed single-cell RNA-sequencing (scRNA-seq) of interzone (IZ) cells and surrounding non-interzone (NIZ) cells (Fig. 3a). A total of 3828 and 8361 cells were obtained from the M/P1 joints of digit III at E14.5 of WT and BDA1 mice, respectively (Supplementary Fig. S6a–f). The combined cell populations were visualized using a tSNE plot (Fig. 3b), and the distributions of IZ and NIZ cells were demarcated using the expression of *Gdf5* as an interzone marker (Fig. 3c). We captured 618 and 1067 IZ (*Gdf5*+) cells, and 3210 and 7294 NIZ (*Gdf5*-) cells from WT and BDA1, respectively. tSNE plots showed overlap between WT and BDA1 cells for both IZ and NIZ, with more separation in the IZ cells (Fig. 3d). The analysis identified 6 sub-populations in the combined IZ cells of WT and BDA1 (Fig. 3e). These sub-populations showed marked differences according to WT or BDA1 origin (Fig. 3f), in terms of their relationship (clusters 2, 3, and 5), and relative proportions of cells (clusters 4 and 6).

Next, we identified the differentially expressed genes (DEGs) for each cluster. Between 3 and 125 signature genes were found for the six clusters (Supplementary Data S2). From a heatmap consisting of the top 10 genes for each cluster and known gene markers, we categorized the clusters into transition cells with mesenchymal characteristics (cluster 1), mesenchymal-like “interzone” cells (cluster 2), two types of ligament-like cells (clusters 3 and 5), chondrocyte-like cells (cluster 6), and actively proliferating cells (cluster 4) (Fig. 3g). Correcting for the total numbers of IZ cells in the two genotypes, relative proportions of “interzone” cells (clusters 1-3) did not differ much but we found disproportionate distributions between WT and BDA1 in some clusters. For example, for BDA1 mice, we noted an enrichment of chondrocyte-like cells (cluster 6) and proliferative cells (cluster 4), but a reduction in one of the ligament-like cells (cluster 3) (Fig. 3h). This is consistent with a change in balance between chondrogenic and non-chondrogenic cells in favor of chondrogenesis, and more proliferating cells could explain in part the expanded interzone.

We then investigated the molecular differences between WT and BDA1 interzone cells (Supplementary Data S3). We subtracted the DEGs between WT and BDA1 in the NIZ cells from those in the IZ cells (Fig. 3i), to derive DEGs in the interzones between the two genotypes. Gene ontology (GO) enrichment analyses showed an enrichment of cell-death-related activities in the genes downregulated in the BDA1 IZ, and an enrichment of chondrogenesis and cell-cycle activities in up-regulated genes (Fig. 3i), indicating the BDA1 interzone contained more dividing chondrocyte-like cells (Fig. 3h). A bubble-plot of the expression levels and percentages of the 15 DEGs related to apoptosis or cell death (Fig. 3j and Supplementary Fig. S6g), shows that many pro-apoptotic genes, such as *Trp53*, are underexpressed in BDA1 compared to WT, in both IZ and NIZ cells. Overall, these genes are more highly expressed in the IZ than in the NIZ, highlighting a functional role of apoptosis in the interzone that is altered in BDA1 joint formation.

To characterize the signals underpinning these changes, we compared DEG between IZ and NIZ cells for each sample (WT or BDA1), with a focus on genes that are more highly expressed in the interzone and/or in BDA1 cells. Two genes were common to all these sets of DEGs, *Gli3*, and *Cdon* (Fig. 3k). Both are related to the Hh pathway: GLI3 is processed upon activation of the Hh signal, converting the GLI3 repressor into a potent transcription activator^65,66^, whereas *Cdon* is a potentiator of Hh signaling^45,67,68^. The distribution of cells with varying expression level of *Cdon* (Fig. 3l) and the percentage of cells expressing *Cdon* are substantially higher in BDA1 IZ and non-IZ cells compared to WT (Fig. 3m). *Gli3* shows a similar expression pattern (Supplementary Fig. S6h). We found that CDON is normally expressed strongly in the developing interzone its expression is elevated in the expanded phalangeal interzones in BDA1 mice (Supplementary Fig. S7). No change in *Gas1* expression was detected (Supplementary Fig. S6i).

### Cell death is involved in phalangeal joint formation and the pathogenesis of BDA1

**Fig. 4:**
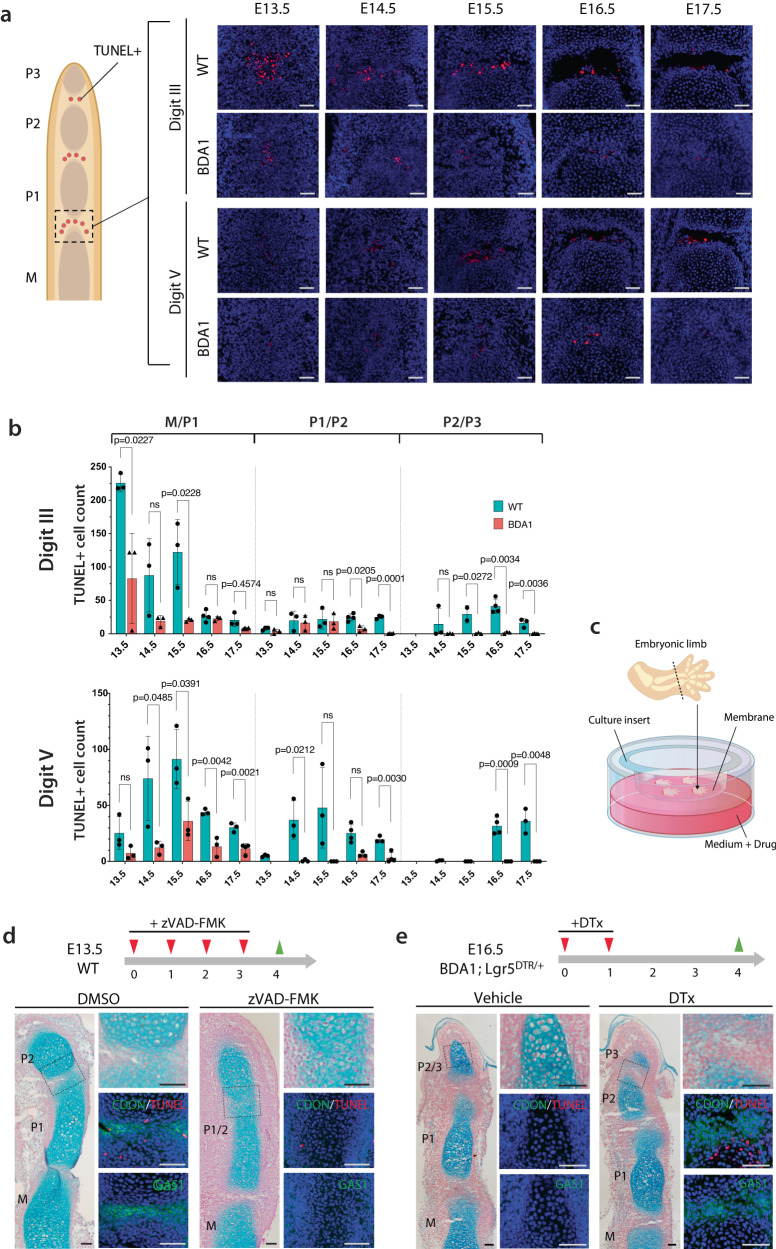
Loss of cell death in the interzone is responsible for BDA1 joint ablation. **a** Representative images of TUNEL staining in M/P1 joints of WT and BDA1 digit III and digit V. Scale bar = 50 µm. **b** Bar charts showing TUNEL-positive counts in the three joints of digits III and V from WT and BDA1. Data were presented as mean values and the error bar indicated standard deviation. Each point represented counts from independent mouse digits. *p*-values were calculated with a two-sided student’s *t*-test. **c** Harvested paws from E13.5 embryos were cultured on inserts with hydrophilic PTFE membrane. Drugs (zVAD-FMK and DTx) were supplied to the culture medium. **d** Pan-caspase inhibitor zVAD-FMK was applied to a culture of E13.5 WT digits for four continuous days. Less TUNEL-positive cells were detected in the joint anlagen. Histology images (stained with Alcian blue and nuclear fast red), and joint markers (GAS1 and CDON) analysis showed P1/2 joint failed to cavitate in the presence of zVAD-FMK. (DMSO *n* = 3, zVAD-FMK *n* = 6). Scale bar = 100 µm. **e** Induction of joint specific cell death in BDA1 digit by supplying DTx to activate *Lgr5*-driven diphtheria toxin receptor (*Lgr5-DTR*) led to formation of P2/P3 joint, indicating by expression of joint markers GAS1 and CDON (*n* = 4). Scale bar = 50 µm. Source data are provided as a Source Data file.

Next, we investigated the involvement of apoptosis in phalangeal joint formation from E13.5 to E17.5, comparing BDA1 to WT mice (Fig. 4a). For the digit III M/P1 joint, apoptotic cells were detected in WT before (E13.5), during (E14.5, E15.5), and at completion (E16.5) of cavitation, fewer apoptotic cells were detected in BDA1 mice (Fig. 4a). Reduced cell death was observed in other digit joints (Supplementary Fig. S8), with few or no apoptotic cells in the P1/P2, P2/P3 interzone regions of BDA1 mice (Fig. 4b). This in vivo finding is consistent with the DEGs in the scRNA-seq and the major GO outcome for reduced apoptosis in the developing BDA1 interzone.

To assess the impact of this reduction in apoptosis in the pathogenesis of BDA1, we studied an ex vivo culture of the digits from E13.5 embryos. These digits grow and interzone formation occurs with the expected proximal to distal progression (Fig. 4c and Supplementary Fig. S9). The interzone of P1/P2 formed on day 4 of culture, indicated by the expression of CDON and GAS1, and the presence of TUNEL-positive cells in the cavitating interzone (Fig. 4d). When we prevented cell death using zVAD-FMK, a pan-caspase inhibitor, the P1/P2 interzone failed to progress by day four, with no detectable expression of CDON or GAS1 at the presumptive interzone region, and little or no evidence of TUNEL-positive cells (Fig. 4d). Thus, cell death is necessary for phalangeal joint development, supporting a loss or reduced cell death in phalangeal interzone formation as a mechanism for the missing P2/P3 joint of digit V in BDA1 mice.

We assessed the possibility of “rescuing” P2/P3 interzone development in digit V of BDA1 mice. We designed a breeding program to produce BDA1 mice carrying a *Lgr5-DTR-GFP* allele^69^, in which GFP is fused to diphtheria toxin receptor (DTR) that marks *Lgr5*-expressing interzone cells with GFP and makes them sensitive to diphtheria toxin (DTx). Culturing E16.5 digits V from this genotype with no drug treatment showed the expected absence of the P2/P3 interzone with no detectable expression of CDON or GAS, or TUNEL-positive cell (Fig. 4e). Treatment with DTx at 0.3 μg/ml for 2 days and allowing the digits to grow for another 2 days led to the formation of the P2/P3 joint, with an expression of CDON and GAS1 and TUNEL-positive cells in the interzone region (Fig. 4e). Importantly, the “rescued” P2/P3 interzone showed evidence for initiation of cavitation, with a distinct interzone space separating the adjacent Alcian blue-positive cartilage anlagen.

### CDON-induced apoptosis suppressed the pathogenesis of BDA1

Reduced cell death and the increase in *Cdon*-expressing cells prompted us to investigate their role in the pathogenesis of BDA1. CDON has been linked to cell death through its potential function as a dependence receptor, whereby, in the presence of a ligand, CDON facilitates Hh signaling, but in the absence of a ligand, CDON can induce cell death^49–51^. We propose that during normal phalangeal joint development, CDON promotes cell death in the interzone where the IHH level is very low. In BDA1, high IHH in the interzone region disrupts this, even though *Cdon* expression is elevated. Thus, we investigated the impact of genetically reducing *Cdon* expression in BDA1^het^ and BDA1 mice on apoptosis in joint formation.

**Fig. 5:**
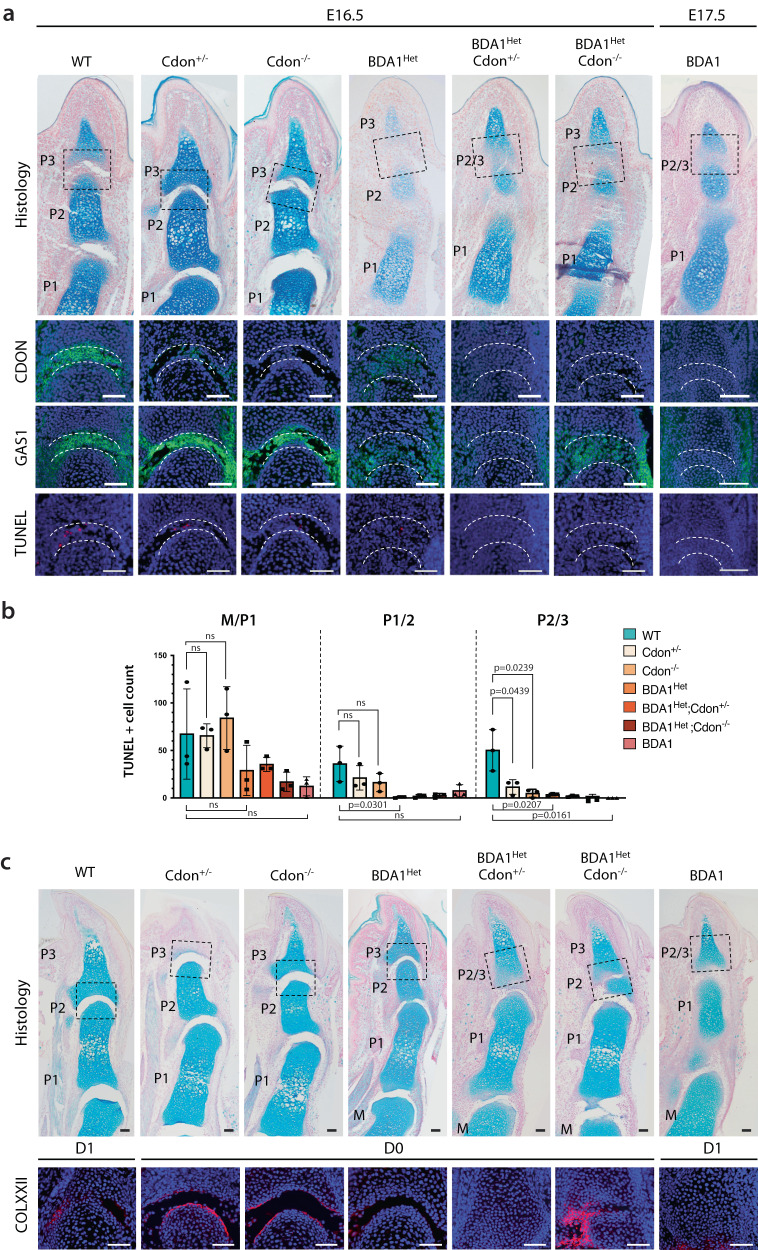
BDA1^Het^ in *Cdon* haploinsufficiency mimics BDA1 ablated P2/P3 joint. **a** Representative histological (Alcian blue stained) images and marker staining of E16.5 and E17.5 digit V of BDA1 and Cdon mutants. Similar abnormal distal joint development was observed in both BDA1^Het^; Cdon^+/-^ double mutant and BDA1. GAS1 and CDON joint markers were not expressed in the distal joint of both genotypes along with loss of TUNEL-positive cells (*n* = 3). Scale bar = 50 µm. **b** TUNEL+ cells of E16.5 digit V were counted and plotted. Data were presented as mean values and the error bar indicated standard deviation. Each point represented counts from one digit (*n* = 3). *p*-values are calculated with a two-sided student’s *t*-test. **c** Histology images and Collagen XXII staining showed loss of P2/3 joint cavitation in digit V of both BDA1^Het^; Cdon^+/-^ double mutant and BDA1 at perinatal stage (*n* = 2). Scale bar = 100 µm. Source data are provided as a Source Data file.

We studied the development of M/P1, P2/P3, and P2/P3 interzones of digit V across relevant genotypes, including WT, Cdon^+/-^, Cdon^-/-^, BDA1^het^, BDA1, BDA1^het^/Cdon^+/-^, and BDA1^het^/Cdon^-/-^. At E16.5 and stage-matched E17.5 BDA1, a P2/P3 interzone can be detected in all genotypes, indicating reducing CDON did not affect the initiation of interzone formation (Fig. 5a). We found no significant differences in the number of apoptotic cells in the M/P1 interzone between WT, Cdon^+/-^ and Cdon^-/-^mice. However, the levels of apoptotic cells were markedly reduced in all genotypes with *E95K* alleles, most strongly in the P1/P2 and P2/P3 interzones, particularly when *Cdon* expression was reduced or inactivated completely (Fig. 5a, b). Thus, there is a direct relationship between the expression of *Cdon* and apoptosis in BDA1^het^ and BDA1 mice, likely due to the abnormally high level of IHH in the developing interzone.

Consistent with the failure of the P2/P3 interzone to progress in digit V of BDA1 mice, the same was observed in BDA1^het^/Cdon^+/-^; the P2/P3 interzone was no longer detectable at D0, while this joint was well formed in BDA1^het^ mice (Fig. 5c). Thus, reducing *Cdon* in BDA1^het^ mice phenocopied BDA1 mice. Surprisingly, complete inactivation of *Cdon* in BDA1^het^/Cdon^-/-^ mice led to cavitation in the P2/P3 interzone as shown by the detection of Collagen XXII, marking the juvenile articular cartilage layer, albeit with some differences compared with the same joint in BDA1^het^ mice (Fig. 5c). This suggests a complex relationship exists and there may be a compensatory mechanism when CDON is absent. A possible candidate is *Gas1* as it is expressed in the developing interzone with a similar but reciprocal expression pattern to *Cdon*, and GAS1 is expressed in the interzones of BDA1^het^/Cdon^-/-^ mice at E16.5 (Fig. 5a).

### IHH suppresses CDON/GAS1-mediated apoptosis

**Fig. 6:**
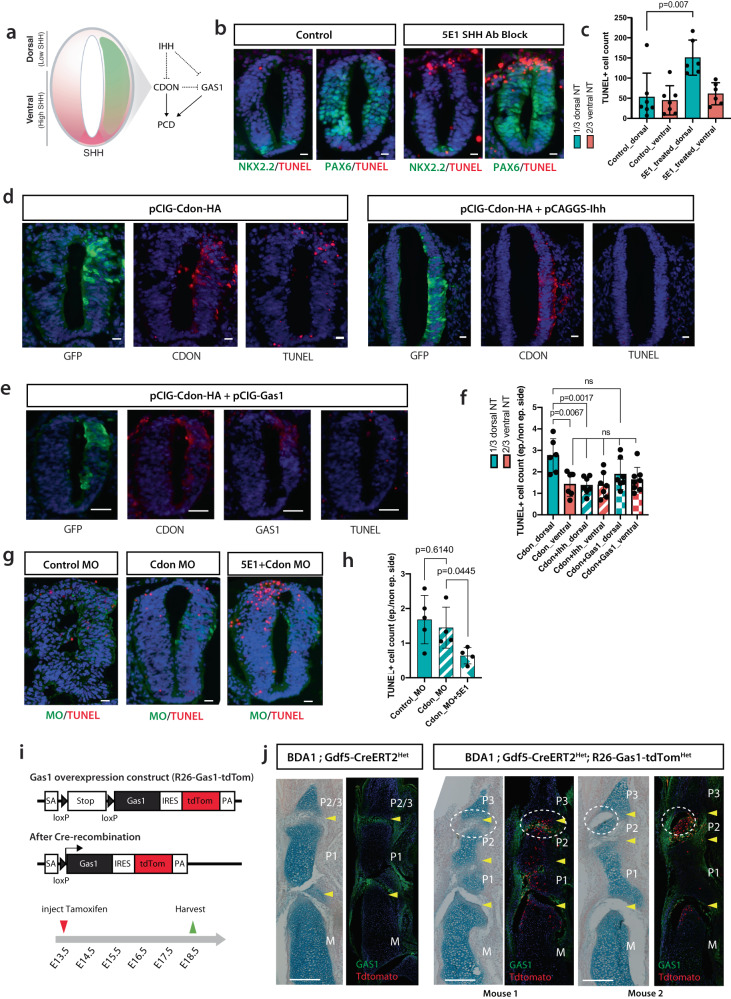
IHH suppressed CDON-mediated apoptosis. **a** Experimental strategy for examining the regulation of programmed cell death (PCD) by IHH, CDON, and GAS1. Dorsal: Dorsal one-third of the neural tube, Ventral: Ventral two-thirds of the neural tube. **b** Treatment of SHH antagonist, 5E1 resulted in increased TUNEL+ cells in the dorsal neural tubes compared to control. **c** Quantification of the number of TUNEL+ cells for **b**). **d** GFP tagged Cdon-expression plasmid was electroporated alone or with Ihh into chick neural tubes. CDON proteins and TUNEL+ cells were detected on sections counterstained with DAPI. **e** Cdon- and Gas1- expression plasmids were co-electroporated into chick neural tubes. **f** Bar chart showing fold-change of TUNEL+ cells counts of the chick neural tubes electroporated with Cdon-, Ihh- and/or Gas1- expression plasmids (**d**, **e**). One-way ANOVA was performed on all groups except the CDON dorsal group. **g** Electroporation of Cdon morpholino (MO) reduced the number of TUNEL+ cells induced by 5E1 treatment in the dorsal neural tubes compared to the untransfected side and neural tubes treated with control MO. Scale bars for **b**, **d**, **e**, **g** = 50 µm. **h** Bar chart showing fold-change of TUNEL+ cell counts of the dorsal chick neural tubes treated with control MO and Cdon MO with/without 5E1 treatment. Each point represented a count from a chick neural tube. **i** Experimental design for *Gas1* overexpression in developing joints. Upon Cre-recombination in the presence of tamoxifen, Gas1, and IRES-tdTomato transgenes were expressed. **j** Overexpression of *Gas1* in joints driven by Gdf5-CreERT2 led to formation of P2/P3 joint in BDA1 mouse (*n* = 2). Yellow arrows indicate the location of joints and a white dotted circle indicates the P2/P3 joint. Scale bar = 200 µm. For the bar chart in **c**, **f**, and **h**, each data point represents an independent biological replicate, bar height indicates mean and error bars indicate standard deviations. *p*-values are calculated with a two-sided student’s *t*-test. Source data are provided as a Source Data file.

To provide mechanistic insight into the roles of CDON and GAS1, and their relationship in regulating apoptosis, we utilized the developing chick neural tube where Sonic Hedgehog (SHH) not only forms a morphogen gradient for dorsal-ventral patterning but also functions as a survival factor for neuroepithelial cells^70,71^(Fig. 6a). The number of apoptotic cells detected was low and no different between the dorsal (low SHH) and ventral (high SHH) neural tubes electroporated with vector alone (Supplementary Fig. S10a, b). Blocking SHH by its antagonist 5E1 resulted in significantly more apoptotic cells in the dorsal region (Fig. 6b, c) where CDON and GAS1 are preferentially expressed^72,73^.

Overexpression of CDON and GAS1 has been shown to induce apoptosis in cancer cell lines^49,74^ Forced expression of CDON in the chick neural tube resulted in an increase in apoptotic cells in the dorsal region where the level of SHH is lowest (Fig. 6d, f), consistent with previous findings^50^ and that co-expression with IHH suppressed the number of CDON-induced apoptotic cells in the dorsal region (Fig. 6d, f). Similar observations were observed when GAS1 was overexpressed alone or in combination with IHH (Supplementary Fig. S11a, b). However, co-expression of CDON and GAS1 did not exacerbate the apoptotic effect compared to CDON or GAS1 alone (Fig. 6e, f and Supplementary Fig. S11a, b), implying there is no additive or synergistic effect.

To investigate whether CDON plays a dominant role in regulating cell viability in the dorsal region, we performed morpholino-mediated CDON knockdown (CDON MO) to reduce the protein level of CDON in this region (Supplementary Fig. S10c). There was no significant difference in the number of apoptotic cells between the transfected and untransfected sides of embryos electroporated with CDON MO and control MO (Ctrl MO) (Fig. 6g, h), thus reducing CDON has no effect on apoptotic outcome. However, CDON plays a dominant role in 5E1-induced apoptosis, as evidenced by a marked reduction of apoptotic cells in the dorsal region of CDON MO electroporated embryos (Fig. 6g, h).

Together, the results suggest that achieving an appropriate balance between SHH survival signaling and the levels of CDON and GAS1 is crucial for regulating cell viability in the chick neural tube. We propose a similar interplay that may exist in the developing joint, whereby CDON may exert a negative effect on GAS1 in the developing joint. A proper balance of these proteins is necessary to achieve the appropriate level of apoptosis required for phalangeal joint cavitation during normal development and in CDON null mice, due to functional compensation by GAS1.

Recognizing the limitation of further exploring this relationship in the chick neural tube and its relevance to the mouse phalangeal joint, we directly assessed the function of GAS1 in the cavitation process. To this end, we generated a mouse conditional for activation of *Gas1* in the *Rosa26* locus (Fig. 6i), to overexpress *Gas1* in interzone cells expressing *Gdf5* from a genetic cross with *Gdf5-CreERT2* mice^75^, upon injection of tamoxifen (TM) to pregnant mice at E13.5 (Fig. 6j). We showed that GAS1 expression in joint formation was activated under this genetic control, using tdTomato as a readout, at the interzone site where *Gdf5* is expressed (Supplementary Fig. S12), and that this rescued the missing P2/P3 joint in digit V of BDA1 mice harvested at E18.5 with clear cavitation and formation of the P2/P3 joint, compared with the control BDA1 mice in the absence of *Gas1* activation, where the P2/P3 joint failed to form (circled regions, Fig. 6j). A possible mechanism is that the presence of more GAS1 “normalizes” the excessive IHH in the P2/P3 joint and the negative effect by CDON, and there are IHH-free GAS1 to induce cell death.

## Discussion

Deciphering the pathogenesis of BDA1 has provided valuable insights into disease mechanism, and conceptual findings in the regulation of Hh signaling and the role of interacting partners working in concert to regulate the strength and range of signaling^76^. Here, we provide evidence for a common outcome of mutations in IHH that cause BDA1, whereby the capacity to activate the Hh signal is reduced but the signaling range is extended due to impaired interactions with co-activating partners (PTCH, CDON, and GAS1), and an inhibitor (HHIP). Importantly, we studied the mechanistic differences between BDA1 individuals with either shortened or missing middle phalangeal bones, or a combination of both. The little finger (digit V) is often the most severely affected, with a missing middle phalangeal bone.

We showed that shortened and missing middle phalangeal bones represent distinct mechanisms. Where the bone is missing, the most distal joint is initiated in development but fails to progress to cavitation. This is associated with an increased concentration of IHH in the developing interzone, which tips the balance against an apoptotic signal required to initiate cavitation, and thus a joint is not formed. Changes in cell death events are supported by single-cell transcriptomics of the developing interzone in a mouse model for BDA1 that highlighted *Cdon* as a key differentially expressed gene. We propose CDON is involved in the apoptotic event and provide supporting evidence from joint development and pathogenesis in BDA1^het^ mice when one copy of *Cdon* is genetically deleted.

In the analysis of *IHH* mutations causing BDA1, we selected a range of mutations around the Ca^2+^ and Zn^2+^ coordination sites that stabilize the structural grooves for interaction with PTCH, CDON, and GAS1. The mutations E95K, D100E, T154I, and R128Q cluster around the interacting grooves, and their mechanistic outcomes are likely to be similar, whereas the homozygous P46L mutation might have a more complex mechanism^57^, in relation to the potential difference in the stabilization effect following the initial interaction of IHH P46L with the partners in the complex^77^.

The variability between shortened and missing middle phalangeal bones in the digits of patients with BDA1 mutations is an interesting characteristic that has not been resolved. Previously, we have shown that the reduced IHH signaling at the digit tip of the BDA1 mouse results in impaired recruitment of surrounding mesenchymal cells into the distal cartilage anlagen prior to the formation of the P2/P3 joint^21^, and with a smaller size cartilage template, a shortened P2 element results when the P2/P3 joint is formed. This can be explained as the P2/P3 joints are formed within the shortened cartilage template^21^. However, this does not explain the absence of the P2 element in digit V.

We showed that a missing joint could occur if the P2/P3 cartilage template was too small for the most distal joint to be initiated, as in the BDB1 mouse with a *Ror2*-null allele^64^. Surprisingly, this is not the case in digit V of the BDA1 mouse, where the P2/P3 interzone is initiated but fails to cavitate and this joint does not form. This is similar to the missing joint phenotype in the *Dsh* mouse where *Shh* is ectopically expressed in the developing P2/P3 joint due to an inversion of the *Shh* locus causing dysregulated expression^18^. The features common to the BDA1 and *Dsh* mice are excess Hh in the developing interzone, and a failure to progress to cavitation.

The last stage in joint formation is cavitation with the physical separation of the cartilage elements and generation of the other joint structures. Very little is known about this cavitation process. Extracellular matrix changes are needed, such as upregulation of hyaluronic acid (HA) and the enzymes that produce it, HA synthase (HAS) and uridine diphosphoglucose dehydrogenase, during cavitation^78,79^. Inactivation of *Has2* in mice resulted in defects in the joint cavity^80,81^. This rapid deposition of HA is thought to alter interactions with the receptor (CD44) or to physically separate cells to create space^79,82^. CD44 in other contexts has roles in cell fate, including activation-induced cell death^83^.

Cell death is detected in certain joints, such as the developing incudomalleolar joint or the temporal-mandibular joints, as part of the mechanism of cavitation^84–86^. The role of apoptosis in limb joint formation is less clear, with opposing findings for knee joint development^39,40,87^. Apoptosis in developing phalangeal joints has been reported^37,38,88^, but it was not known whether it was necessary for cavitation. Here, we provide direct evidence linking cell death and progression to cavitation in phalangeal joints from E13.5 to E17.5. The amount of cell death is significantly reduced in the BDA1 mouse in all developing interzones in the digits but is sufficient to allow progression to cavitation and joint formation, except in the P2/P3 interzone in digit V, where little or no cell death is detected, and cavitation does not occur. Stimulation of cell death by treatment with diphtheria toxin rescues the missing P2/P3 joint in BDA1 mice. Conversely, inhibiting cell death impaired progression to cavitation in digits of WT mice.

Several interacting partners in the Hh signaling pathway, including PTCH1, CDON, and GAS1, have characteristics of dependence receptors^48,49,55^. Dependence receptors, of which there are about 20 members with diverse features^89^, can transduce both innate signaling through ligand binding and an alternative cell death pathway in the absence of ligand. Thus, the discovery of *Cdon* as a key DEG in the single-cell transcriptomic data prompted us to investigate its role in the missing joint phenotype in digit V of BDA1 mice. While the DEG data indicated an increase in *Cdon* expression in BDA1 interzones, inactivation of one *Cdon* allele in BDA1^het^ mice phenocopied BDA1 mice with a missing digit V P2/P3 joint, indicating a role for CDON in the pathology. This was associated with a further reduction in cell death, which suggests a link between *Cdon* expression and CDON concentration and cell death. However, when *Cdon* was completely inactivated in BDA1^het^ mice, the P2/P3 joint progressed to cavitation, suggesting a compensatory mechanism that is suppressed when *Cdon* is expressed. *Gas1* can induce apoptosis when overexpressed in the chick neural tube and we found that overexpression of *Gas1* in *Gdf5*-expressing interzone cells rescued the BDA1 P2/P3, allowing progression to cavitation and joint formation.

**Fig. 7:**
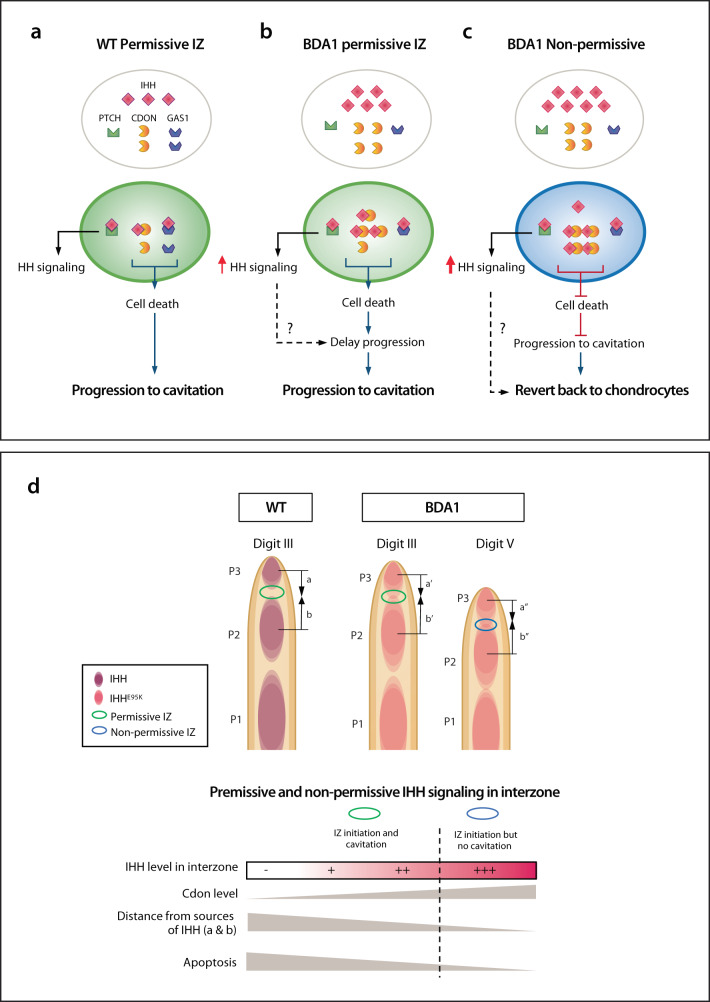
Model of permissive and non-permissive environment for joint formation and implication to the pathogenesis of BDA1. **a** A permissive interzone (IZ) environment with a balanced ratio of IHH ligands and interacting partners such that there are ligand-free CDON to induce apoptosis leading to cavitation and thus joint formation. **b** A permissive interzone environment in BDA1 mice where there is an increase in the level of IHH and interacting partners; but the balance of IHH ligand and partners still allows sufficient ligand-free CDON to induce apoptosis for cavitation to proceed; albeit with a slight delay due to enhanced IHH signaling. **c** A non-permissive interzone environment in BDA1 mice where the increase in the level of IHH and is above that of the partners, and there is no ligand-free CDON to induce apoptosis for cavitation to proceed, and a joint is not formed. **d** Model in the context of distal joint formation illustrating the normal molecular level of the key signals and events leading to an increased IHH level in the developing P2/P3 joint, and the balance between the concentration IHH and CDON to ensure a threshold level ligand-free CDON for apoptosis to take place, defining the boundary between permissive and non-permissive interzone environment for cavitation to be initiation and progression in joint formation.

To place our findings in the context of joint formation, we propose a model in which the progression of an interzone to cavitation requires a permissive environment in the inner region of the interzone, with a low concentration of IHH and a high concentration of its interacting partners (PTCH, CDON, GAS1) (Fig. 7a). Thus, there are sufficient unbound partners (CDON and GAS1) to induce cell death and cavitation can proceed (Fig. 7a). When CDON is present, the apoptotic function of GAS1 is suppressed, but in its absence, GAS1 can compensate for CDON. GAS1 is a GPI-anchored membrane protein that does not have an intracellular “death activating” domain, so to act as a dependence receptor, it would require a partner^90,91^. In BDA1 mice, even though there is a high concentration of IHH within the interzone, it is counterbalanced by an increase in *Cdon* expression so that there is sufficient free CDON to induce cell death, and a joint is formed albeit with some delay (Fig. 7b). However, the more distal P2/P3 interzone in digit V does not permit cavitation to proceed. We propose this interzone experiences the highest concentration of ectopic IHH, which leaves little or no free CDON, so the apoptotic function of CDON is suppressed and, in combination with increased IHH signaling as a chondrogenic signal, the interzone cells revert to chondrocytes and a joint is not formed (Fig. 7c). In the *Dsh* mouse, this would be the case for all the digits as the level of SHH in all the P2/P3 interzones across digits II-V would be sufficiently high to suppress apoptosis. It would be interesting to determine the role of CDON and GAS1 as dependence receptors in joint formation, and of downstream partners to provide conceptual insights into the induction of cell death that involves the recruitment and activation of pro-apoptotic effectors such as caspase-9 or the serine-threonine kinase DAPK (death-associated protein kinase)^48,92,93^.

In the overall perspective of digit pathogenesis in BDA1, we have extended our molecular understanding of the mutations as a consequence of reduced IHH signaling capacity but increased range to explain the reduced size of distal digit bones^21^, to the occurrence of interzone environments permissive and non-permissive for cavitation in the joint-forming process (Fig. 7b, c). As digit V is the smallest digit and the distance from the proximal (cartilage element) and distal (digit tip) sources of IHH is small, this P2/P3 interzone receives the most IHH (Fig. 7d). Thus, the threshold for a permissive versus a non-permissive interzone environment is a combination of the distance from the sources of IHH, the level of IHH and its interaction with partners with dependence receptor functionality defining cell death events (Fig. 7d).

## Methods

### Amino acid sequences, multiple alignments, and protein structure

Mouse (P97812), human (Q14623), and chicken (Q98938) IHH protein sequences were obtained from Uniprot, then aligned and visualized using Pfaat 2.0.130 (PMC2092438). Annotated residues for partner binding and interactions were obtained by manual literature searches. 3D protein structures were obtained from the US Research Collaboratory for Structural Bioinformatics Protein Data Bank (RCSB PDB) database for MuShh-HuHHIP (ID:2wfx), MuShh-HuCDON (ID:3d1m) and HuSHH-HuPTCH1 (ID:6dmy). Protein interactions were visualized and analyzed by PyMOL Ver2.4.0. (The PyMOL Molecular Graphics System, Version 2.0 Schrödinger, LLC.)

### Chick embryos and in ovo electroporation

White leghorn fertilized chick eggs from Jinan Poultry Co. (Tin Hang Technology) were incubated at 38.5 °C and 65% humidity in a humidified incubator. Embryos were checked every 6 h to ensure good condition and normal embryo development. Embryos were staged according to Hamburger and Hamilton (HH) stages^94^. The embryos were euthanasia immediately with 4% PFA once were found unhealthy during the development. The Committee on the Use of Live Animals in Teaching and Research at the University of Hong Kong approved the chick experimentations (CULATR No: 5969-21). Chicken neural tube electroporation was performed as previously described^95^. Plasmid DNAs or morpholinos were injected into the lumen of neural tubes at HH10-11, followed by electroporation using a BTX electroporator delivering five 50 ms pulses at 33V. Electroporated embryos were allowed to develop for 24 h post-transfection before being processed for Western blotting to examine CDON expression levels, immunofluorescence for dorsal-ventral markers on transverse sections, and TUNEL assay to detect apoptotic cells.

Morpholinos (MO) for Cdon (Cdon MO) and corresponding control (Ctrl MO) tagged with 3’ fluorescein were obtained from Gene Tools with the following sequence:

Cdon- MO: 5’ CCGGACAGCAGGCACCACAACGG 3’

Ctrl- MO: 5’ CCTCTTACCTCAGTTACAATTTATA 3’

The final molar concentration of each morpholino oligonucleotide was 0.75 mM mixed with V5-EGFP as the carrier DNA for in ovo electroporation. Chick embryos at HH10-11 or transfected with Cdon MO or plasmid DNAs were treated with 5E1 antibody. After removing the vitelline membrane, we applied 2 µl of the 5E1 antibody to the surface of the dorsal neural tube. After 8 h incubation, treated embryos were harvested and dissected in ice-cold PBS before fixing in 4% (w./v.) PFA in PBS for 2 h on ice. The fixed embryos were cryoprotected with 30% (w./v.) sucrose in PBS and sectioned at 12 µm thickness using Thermo Cryostar NX50.

### Chicken neural tube luciferase assay

Luciferase assay was conducted by the Promega Dual-luciferase Reporter Assay System (Promega E1960) according to the manufacturer’s manual. Gli1-firefly luciferase and Renilla DNA reporter constructs were co-electroporated with target plasmids into developing chick embryos. The neural tube at the heart level was dissected and dissociated, and the activity was recorded according to the luciferase manual. Luciferase signals were detected by SpectraMax Microplate Reader (Molecular Devices). The ratio between the reporters was titrated at Hedgehog signaling saturation to optimize the most sensitive and linear range of conditions.

### Vectors and molecular cloning

pCAGGS-Ihh (N-terminus of Ihh only) and pCAGGS-IhhE95K were generated as previously described^21^. IhhD100E was generated from pCAGGS-Ihh via site-directed mutagenesis and IhhP46L, IhhR128Q, and IhhT154I via overlapping-PCR cloned into pCAGGS-Ihh. Site-directed mutagenesis^96^ and overlapping mutagenesis^97^ were performed as described. PCR of both mutagenesis methods was performed with Phusion polymerase (NEB N0530) under the standard Phusion program with respective PCR and sequencing primers (Supplementary Data S1). pGli1-LucII containing octameric Gli1 binding site driven firefly luciferase plasmid (8×3’Gli-BS-delta51-LucII) (RIKEN RDB68061)^98^ as well as mammalian expression plasmids pCAGGS-Hhip, pCIG-Gas1 and pCIG-Cdon-HA were obtained from Prof. James Briscoe, Crick Institute. All plasmids were transformed into *E. Coli* XL10-Gold. Miniprep (Qiagen 27104) and maxiprep (Favorgen FAPDE 003) were performed according to the manufacturer’s instructions.

### Mouse strains

*Ihh*^*E95K*^ (BDA1)^21^ was maintained in a mixed ICR/C57BL/6 background. *Lgr5*^*eGFP-IRES-CreERT2*^ (Lgr5-GFP)^99^, *Lgr5*^*DTR-eGFP*^ (Lgr5-DTR-GFP)^69^ and *Gdf5*^*CreERT2*^ (Gdf5-Cre)^75^ were maintained in C57BL/6 background*. Cdon*^*LacZ*^ (Cdon^-/-^)^100^ was maintained in the 129/SV background. ROSA-Gas1-tdTomato (R26-Gas1) overexpression mice were generated by morula aggregation of C57BL/6 background. Compound mutants from parents with different backgrounds were bred for at least 3 generations before analysis to ensure similar genetic backgrounds among generations. WT mice were harvested as littermates of respective mutants. Mice were bred under the AAALAC International accredited program at the Center for Comparative Medicine Research, HKU, under specific pathogen-free conditions. Mice were housed in a controlled environment (20–22 °C and 69–71% humidity with 12 h light/dark cycle) and fed ad libitum with standard chow (LabDiet, #5053). Mice were monitored for physical changes indicating a deterioration of health, weight changes, and changes in breathing patterns, and distressed mice were euthanized by an overdose of pentobarbital. All mouse experimentations were approved by the Committee on the Use of Live Animals in Teaching and Research of the University of Hong Kong (CULATR No: 4494-17). Euthanasia was performed with an animal license under the regulation of the Department of Health, HKSAR. Sex was not a selection criterion and mice of both sexes were used in all studies concerned. Reporting of in vivo experiments is in accordance with the ARRIVE guidelines.

### Generation of R26-Gas1 mice with a conditional activation allele for *Gas1*

Full-length Gas1 fragment was amplified by PCR using primers 5’-CTCGAGATGCTAGCCGCGCTGC-3’ and 5’-GGATCCCTACAAGTGTGACCCGAGC-3’ with C57BL/6 genomic DNA as template. A 1036 bp amplicon was cloned into a pBS-IRES-TdTomato cassette in pBigT (#21270) and then into pROSA26-PA (#21271), yielding a ROSAarm-Gas1 plasmid (ROSAleft-LoxP-3xPolyA-LoxP-Gas1-IRES-TdTomato-ROSAright). Mouse ESC line, L4, maintained in 2i/LIF medium on gelatin coating^101,102^ were co-transfected with ROSA-Gas1 plasmid and PX459 (#62988) using LIPO2000 (Thermo Fisher) according to manufacturer’s protocol. Ninety-six transfected G418-resistant clones were genotyped, and correctly integrated clones were assessed for tdTomato expression and copy number analyzed by qPCR. One clone was selected for the generation of chimeras and cross-breeding to produce heterozygous mice containing the conditional allele using an established protocol^103^.

### DNA extraction and mouse genotyping

Ear punch biopsies were digested in TE buffer (pH7.5) containing 0.5% (w./v.) Sodium dodecyl-sulfate (SDS) and 1 mg/ml Proteinase K (Thermo EO0491) at 65 °C overnight. DNA was purified by using Phenol-chloroform (Thermo 15593031) extraction followed by ethanol precipitation. Genotyping primers and amplicon sizes are listed in Supplementary Data S1. Genotyping PCR of BDA1, Lgr5-GFP, Lgr5-GFP-DTR, ROSA-Gas1-tdTomato, and Gdf5-CreERT2 alleles were performed using standard Taq master mix (NEB M0273), while *Cdon*^-/-^ allele was genotyped using KAPA2G Robust Hotstart Readymix (Roche 2GRHSRMKB) according to manufacturer’s protocol. An additional 10 touchdown cycles of −0.5 °C/cycle were performed for all genotyping PCR reactions.

### Mouse conditional activation of *Gas1* by Tamoxifen

Tamoxifen (Sigma T5648) was dissolved in corn oil (Sigma C8267) at 20 mg/ml concentration at 37°C and gently shaken overnight. The prepared solution was stored at 4°C for no more than 1 week. Tamoxifen was injected intraperitoneally into pregnant mice at 200 mg/kg body weight and sacrificed at an appropriate gestation stage by cervical dislocation. Fetuses and neonates ( < 10 days) were sacrificed by decapitation.

### Single-cell transcriptome analysis

Digit III M/P1 interzones (GFP-labeled) at E14.5 with minimum surrounding tissues (GFP negative) were harvested with the aid of Lgr5-GFP in WT (Ihh^+/+^; Lgr5^GFP/+^) and Lgr5-DTR-GFP in BDA1 (Ihh^E95K/E95K^/Lgr5^DTR-GFP/+^). A total of 14 joints from two litters from the same father of each respective genotype were harvested. Dissected tissues were dissociated with 0.02% (w./v.) collagenase P (Roche 11213865001) for 45 min at 37 ^°^C, followed by straining through a 40 μM cell strainer. Single cells were encapsulated using 10X Genomics Chromium Single-Cell Chip (10x genomics) with Chromium Single-Cell 3’ Reagent Kits v2 according to manufacturer’s instructions, performed by Center of PanorOmic Sciences, HKU. Libraries were sequenced by HiSeq1500 (Illumina) and NovaSeq6000 (Illumina).

The raw sequencing data was processed with CellRanger (version 3.1.0, 10X Genomics Inc.) and aligned to the mouse genome (mm10). For the WT sample, a total of 3846 cells, with a median of 2020 genes per cell and a sequencing saturation of 85.8%, were detected. For the mutant sample, a total of 8394 cells, with a median of 1318 genes per cell and saturation of 88.2%, were detected. The two samples were first separately analyzed. Gene variability analyses showed that *Gdf5* is among the top variable genes in both samples (Supplementary Fig. S6a, b). Seven clusters of cells were identified in the WT (Supplementary Fig. S6c, d). Coincidentally, an identical number of clusters of cells were found in the mutant (Supplementary Fig. S6e, f). To reduce potential batch effects when analyzing the two samples in combination, first, a canonical correlation analysis (CCA, using top 50 canonical vectors) with the Seurat package (version 3.0.0)^104^ was applied before performing dimension reductions (tSNE or UMAP). Second, in calculating the DEGs, we used the Wilcoxon Rank Sum test with the Benjamini & Hochberg method for correcting multiple tests. A gene with a corrected *p*-value (FDR) < 0.05 and a log(fold-change) >0.5 or <−0.5 is considered a DEG. We then subtracted those that were also DEGs between WT and mutant non-interzone, by assuming that those shared by both interzone and non-interzone are probably due to batch effects or genetic backgrounds. In all, 6 clusters of cells were identified in the combined interzone data (Fig. 3d–f) and annotated with biological identities based on their respective signatures (DEGs of each cluster versus all remaining cells) (Fig. 3g). The normalized distribution (Fig. 3h) of cells across the genotypes for cluster $i$, were calculated as: $({n}_{i}/N)/({n}_{i}/N+{m}_{i}/M)$ and $({m}_{i}/M)/({n}_{i}/N+{m}_{i}/M)$, where ${n}_{i}$ and ${m}_{i}$ are the numbers of WT and mutant cells in cluster $i$, respectively; and $N$ and $M$ are the total numbers of WT and mutant interzone cells, respectively. Gene-ontology analyses were performed on GSEA^105^.

### *Lgr5*^*DTR/+*^ ex vivo paw/digit culture

E13.5 WT or E16.5 BDA1; *Lgr5*^*DTR/+*^ embryonic paws with skin on the ventral side removed were cultured ventral side down on culture inserts (Millicell PICM0RG50, 0.4 μM hydrophilic PTFE membrane) on a 6-well plate. Each insert contains 4-5 paws and wells were filled with a minimal volume of Gibco^TM^ BGJb medium (Thermo 12591038) just sufficient to moisten the insert membrane. Caspase inhibitor zVAD-FMK (R&D FMK001) and Diphtheria toxin (DTx) (Sigma D0564) were diluted to 20 mM and 2 mg/ml, respectively, according to the manufacturer’s protocol. Culture media was changed daily.

### Mouse tissue processing

Mouse embryonic tissues were fixed in 4% (w./v.) PFA in PBS overnight at 4 ^°^C with gentle rolling. Peri- and postnatal mouse tissues were decalcified in 0.5 M EDTA (pH 8.0) at 4 ^°^C overnight after fixation. After fixation, all tissues were infiltrated with 30% (w./v.) sucrose in PBS overnight at 4 ^°^C with gentle rolling and embedded in OCT medium (Leica 14020108926) for cryo-sectioning. Cryo-sectioning was sectioned at 5 µm thickness using Cryostat Leica 3050 S and Thermo Cryostar NX50.

### Immunohistochemistry, in situ hybridization, TUNEL, and Alcian blue staining

For immunohistochemistry, rehydrated cryosections were permeabilized in 0.1% (v./v.) Tween-20 in PBS for 10 min and subsequently blocked with 3% (w./v.) BSA in PBS before incubating with primary antibodies overnight at 4 ^°^C. Mouse anti-NKX2.2 (DSHB 74.5A5; 1:100 dilution), mouse anti-PAX6 (DSHB Pax6; 1:20 dilution), goat anti-CDON (R&D Systems AF2429; 1:200 dilution), goat anti-GAS1 (R&D Systems AF2644; 1:200 dilution) and sheep anti-EGFP (AbD Serotec 4745-1051; 1:1000 dilution) were used for chicken neural tube analysis. Rabbit anti-IHH (Novus NB110-57122; 1:250 dilution), rabbit anti-HHIP (Bioss 12316-R; 1:200), goat anti-GFP (Abcam ab6673; 1:500 dilution), guinea pig anti-COLXXII (gift from Dr. Manuel Koch; 1:1000 dilution), goat anti-CDON (R&D Systems AF2429; 1:100 dilution) and goat anti-GAS1 (R&D Systems AF2644; 1:100 dilution) were used for mouse analysis. Signals were detected using corresponding Alexa® Fluor secondary antibodies for 1 h at room temperature in 1:1000 dilution, namely anti-sheep 488 (Thermo, A11015), anti-mouse 594 (Thermo A11005), anti-goat 488 (Abcam ab150141), anti-goat 594 (Abcam ab150132), anti-guinea pig 488 (Jackson ImmunoResearch 706-545-148), anti-guinea pig 594 (Jackson ImmunoResearch 706-585-148) and anti-goat 637 (R&D Systems NL002; 1:200 dilution).

In situ hybridization for *Gdf5* was performed using [S^35^] uridine triphosphate-labeled riboprobes as previously described^21^. TUNEL assay was performed before immunofluorescent detection of CDON and GAS1 for co-localization. TUNEL assay was performed according to the manufacturer’s instructions (Sigma 12156792910).

For histology, wax sections after rehydration or cryosections after OCT removal were briefly rinsed in water and then stained in 0.015% (w./v.) 8GX Alcian blue solution for 10 min (Sigma A5268), followed by gentle rinsing under tap water and subsequent nuclear fast red staining for 30 s (Vector H-340). Sections were then washed using tap water, dehydrated twice with absolute ethanol for 5 min each, followed by two xylene washes for 5 min each, then mounted with DEPEX mounting medium (Sigma 06522).

### Western blot analysis

Ten well-transfected chick neural tubes at the trunk level with either Ctrl-MO or Cdon MO 12 hpt were excised and subsequently dissociated in 0.05% trypsin (Gibco). The resulting single cells were sorted using BD FACS Aria^TM^ SORP and transfected GFP-positive cells were lysed on ice. The protein lysates were then resolved on a 6% sodium dodecyl-sulfate polyacrylamide gel electrophoresis (SDS-PAGE) gel, transferred to a **poly(vinylidene fluoride)** membrane (Bio-Rad), and blocked with 5% (w./v.) BSA (Sigma-Aldrich). The membrane was immunoblotted with antibodies against β-tubulin (1:2000, Cell signaling technology) and CDON (1:500, Thermo Fisher), and the signal was visualized by chemiluminescent (ECL, Advansta).

### Imaging

Fluorescent images were acquired using Olympus BX51 and BX53 fluorescence microscope with SPOT camera and Hamamatsu digital camera C11440, respectively. Higher-resolution fluorescent images were acquired using Zeiss LSM 880 with Ariyscan 2 inverted confocal microscope. Confocal microscopy images were processed by the ZEN software. For images with multiple staining, different fluorescent channels were merged as indicated in the figure. Histological images were acquired with a Zeiss Axioplan 2 microscope with a SPOT camera and an Olympus BX51 microscope with a Promicam USB 3.0 Digital Camera. Constant imaging parameters were applied to images of the same batch. The brightness, contrast, and color of some images were slightly adjusted with the “brightness/contrast”, “hue/saturation”, and “color balance” functions in Adobe Photoshop CS. Some images in Fig. 6 were flipped horizontally to keep consistent with the side of electroporation. No further modifications were made.

### Statistics

Statistical analysis was performed using GraphPad Prism 8.4.3 (GraphPad Software, Inc., CA, USA). The exact sample size (*n*) for each experimental group is represented as individual dots in the graphs. Measurements were taken from distinct mice or chickens. Error bars indicated standard deviation. *p*-values for statistical results are indicated within the graphs. For *Gli1*-firefly luciferase assay, the signal was calculated based on $\frac{{sample}\,({electroporated}/{non}-{electroporated})}{{mean\; of\; control}\,({electroporated}/{non}-{electroporated})}$. Fold-change is calculated based on the mean of samples. For chicken neural tube NKX2.2 and PAX6 quantification, the signal was calculated by $\frac{{Staining\; intensity}\,\left({Integrated\; density\; of\; electroporated\; side}\right)-{background\; intensity}}{{area\; of\; electroporated\; side}}$, where background intensity is the multiple of the mean gray value of the non-electroporated side and the area of the electroporated side. All measurements were performed on ImageJ (v1.53) using the in-built analyze>measure function on ROI. For the TUNEL assay, individual TUNEL signal without overlapping other TUNEL fluorescence was labeled as 1 positive count. For the TUNEL assay regarding mouse digit apoptosis, statistical significance was determined using an unpaired two-tailed Student’s *t*-test comparing the number of TUNEL+ cells in WT and BDA1 for each time point. For apoptotic studies utilizing the chicken electroporation system, TUNEL+ cell count was calculated from 8 sections for each chicken neural tube, and the fold-change was calculated based on $\frac{{TUNEL}+{cells\; at\; electroporated\; side}}{{TUNEL}+{cells\; at\; non}-{electroporated\; side}}$. Statistical significance was determined using an unpaired two-tailed Student’s *t*-test.

### Reporting summary

Further information on research design is available in the Nature Portfolio Reporting Summary linked to this article.

### Supplementary information

Supplementary Information

Description of Additional Supplementary Files

Supplementary Data S1-S3

Reporting Summary

### Source data

Source Data

## Data Availability

The raw data of the scRNA-seq used in this study was deposited on the NCBI GEO database (accession number: GSE183253). The processed scRNA-seq data is also available on an interactive web interface at: https://www.sbms.hku.hk/dclab/BDA1. Source data are provided in this paper.
