# Supplementary material for: Suppression of apoptosis impairs phalangeal joint formation in the pathogenesis of brachydactyly type A1
Source: Nat Commun. 2024 Mar 12;15:2229. doi: 10.1038/s41467-024-45053-0 (PMC10933404; doi:10.1038/s41467-024-45053-0)
Supplement: Supplementary file 1 — Supplementary Information [file 41467_2024_45053_MOESM1_ESM.pdf]

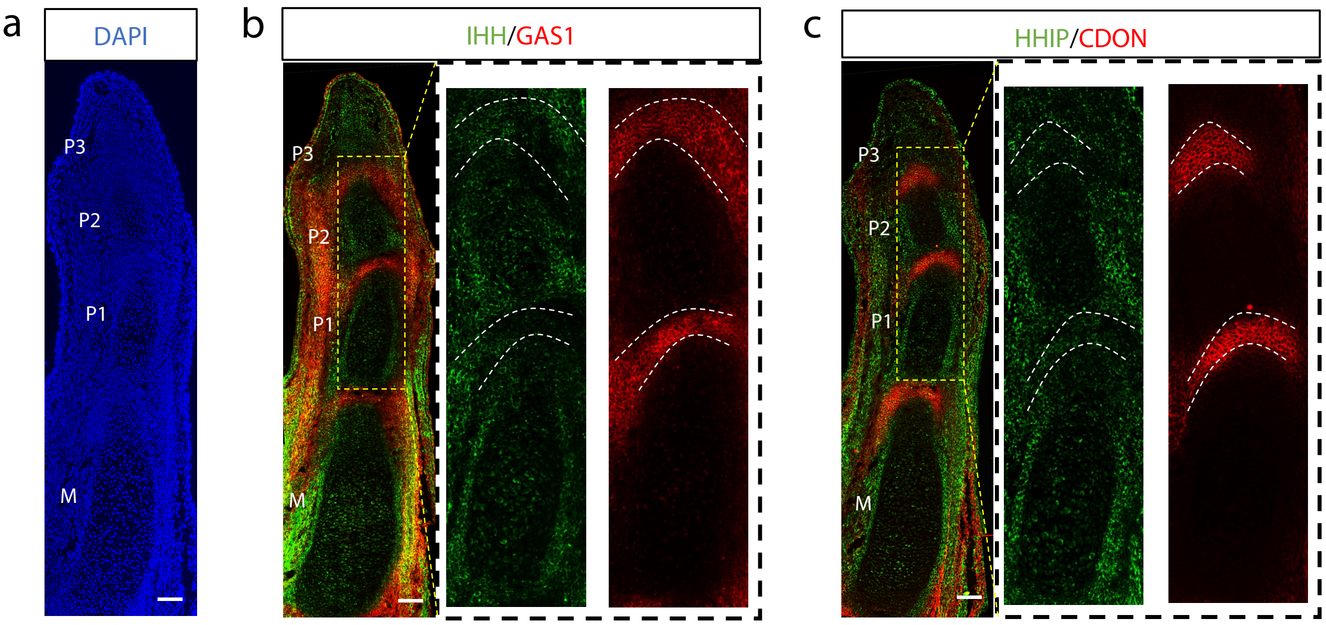

Supplementary Figure S1. **Expression of IHH and its interacting partners in mouse E14.5 developing digit.** Fluorescence immunostaining of **a** DAPI, **b** IHH and GAS1 and **c** HHIP and CDON on sagittal sections of WT digit **III**, showing CDON and GAS1 were expressed specifically in the developing digit interzone, IHH and HHIP were detected along the anlage including the interzone. Scale bar = 100 μm.

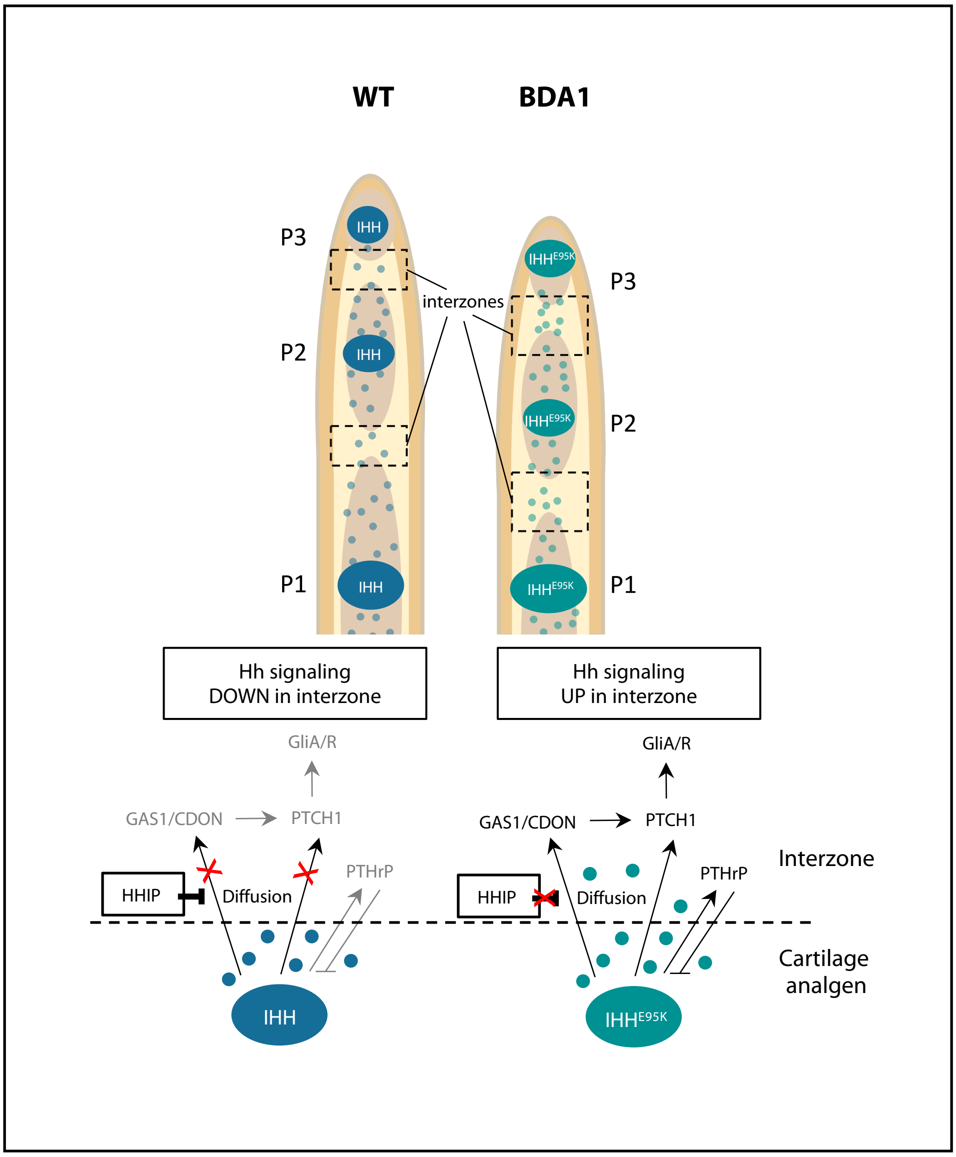

Supplementary Figure S2. **IHH signalling field and interacting partners in developing digits.** In BDA1, IHH diffusion field is enlarged due to the reduction of HHIP inhibition. The elevated amount of IHH^{E95K} in interzone promotes Hh signaling and activates GLI1 effectors by increased PTCH1/GAS1/CDON receptor binding. Distal Hh also increases PTHrP signaling and hence further negatively regulates IHH expression.

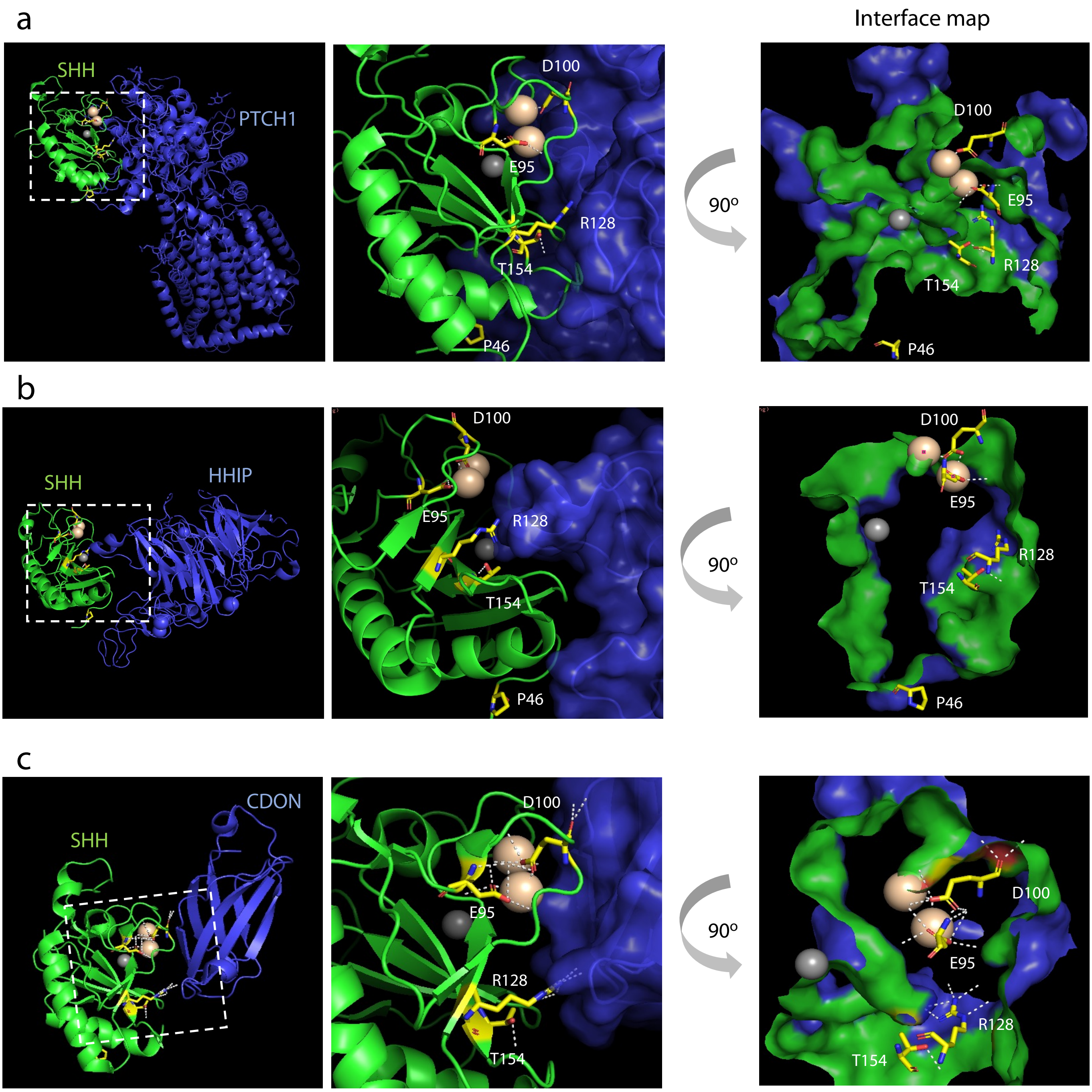

Supplementary Figure S3. **Structural mapping of BDA1 and ACFD mutation sites on SHH in relation to binding partners.** BDA1 and AFCD mutation sites were mapped on SHH protein while interacting with HH partners **a** PTCH1, **b** HHIP, and **c** CDON, visualized by PyMOL. Human or mouse SHH is represented in green with mutations highlighted in yellow, bound partners are in blue. Zn^{2+} and Ca^{2+} ions are displayed as grey and brown spheres respectively. BDA1 mutation sites (E95, D100, R128 and T154) are localized at the Ca^{2+} and Zn^{2+} interacting groove with D100 and E95 directly interacting Ca^{2+}. R128 and D100 and T154I is in close proximity to PTCH1, HHIP and CDON. Space-filled interface map (PyMOL: InterfaceResidues) demonstrated the direct contribution of the 3 mutations as interacting residues, while E95 regulates the 2 Ca^{2+} ions responsible for all interactions. P46 is situated outside the main interaction groove but is still in proximity to PTCH1 and HHIP but not CDON.

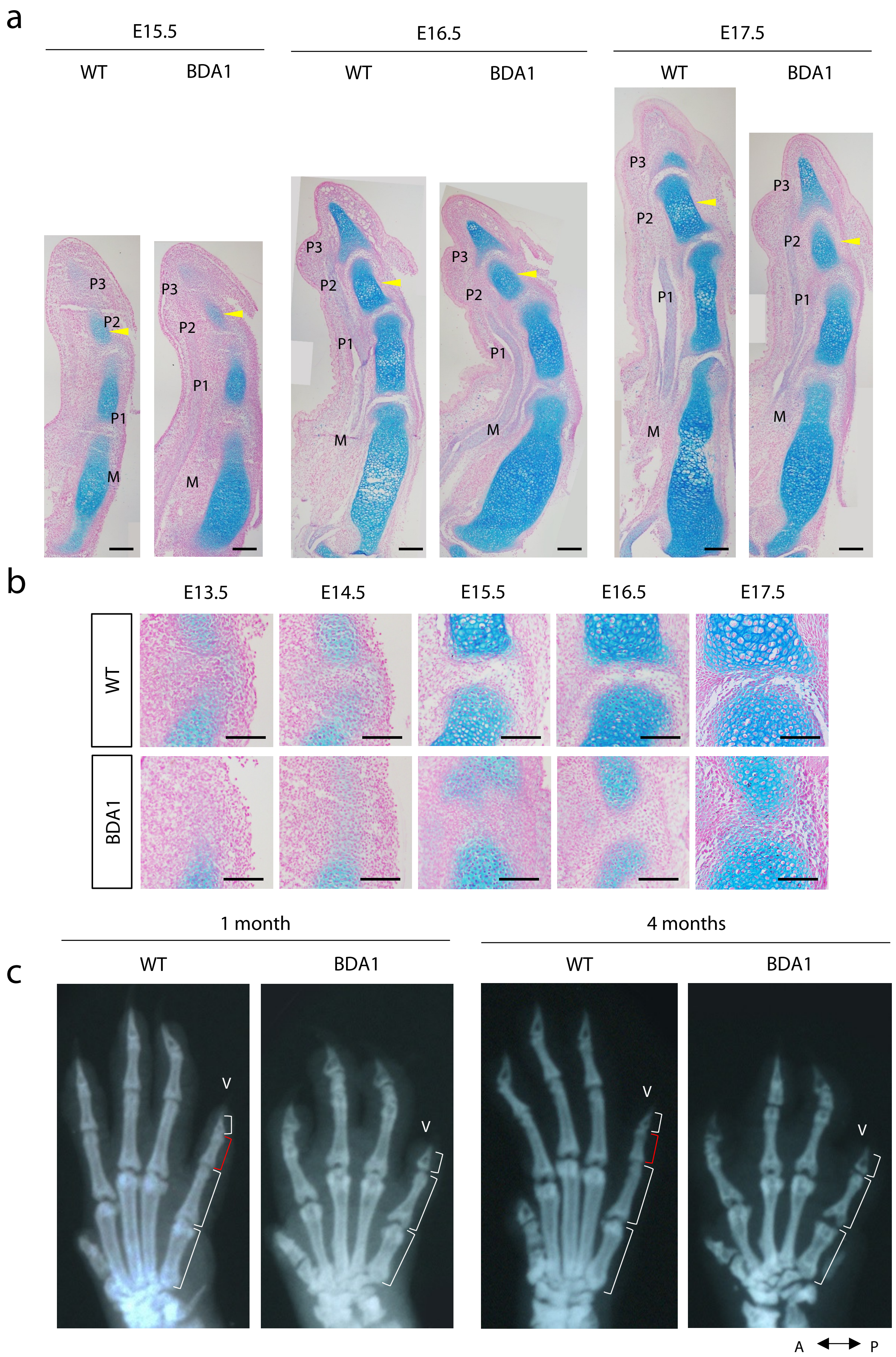

Supplementary Figure S4. **Detailed analysis of BDA1 phenotype in pre- and postnatal stages. a** Alcian blue and nuclear fast red histological staining of WT and BDA1 digit III, showing joints were cavitated and anlage were separated of all BDA1 digit III joints but shortened middle anlagen P2 (yellow arrowheads) (n = 2). Scale bar = 200 μm. **b** Alcian blue and nuclear fast red histological staining of WT and BDA1 digit V P1/2 joint, highlighting approximate 1-day delay in development within the BDA1 joints (n = 3). Scale bar = 100 μm. **c** X-Ray radiographs of postnatal WT and BDA1 demonstrating the absence of BDA1 distal joint was maintained in adulthood (n = 3).

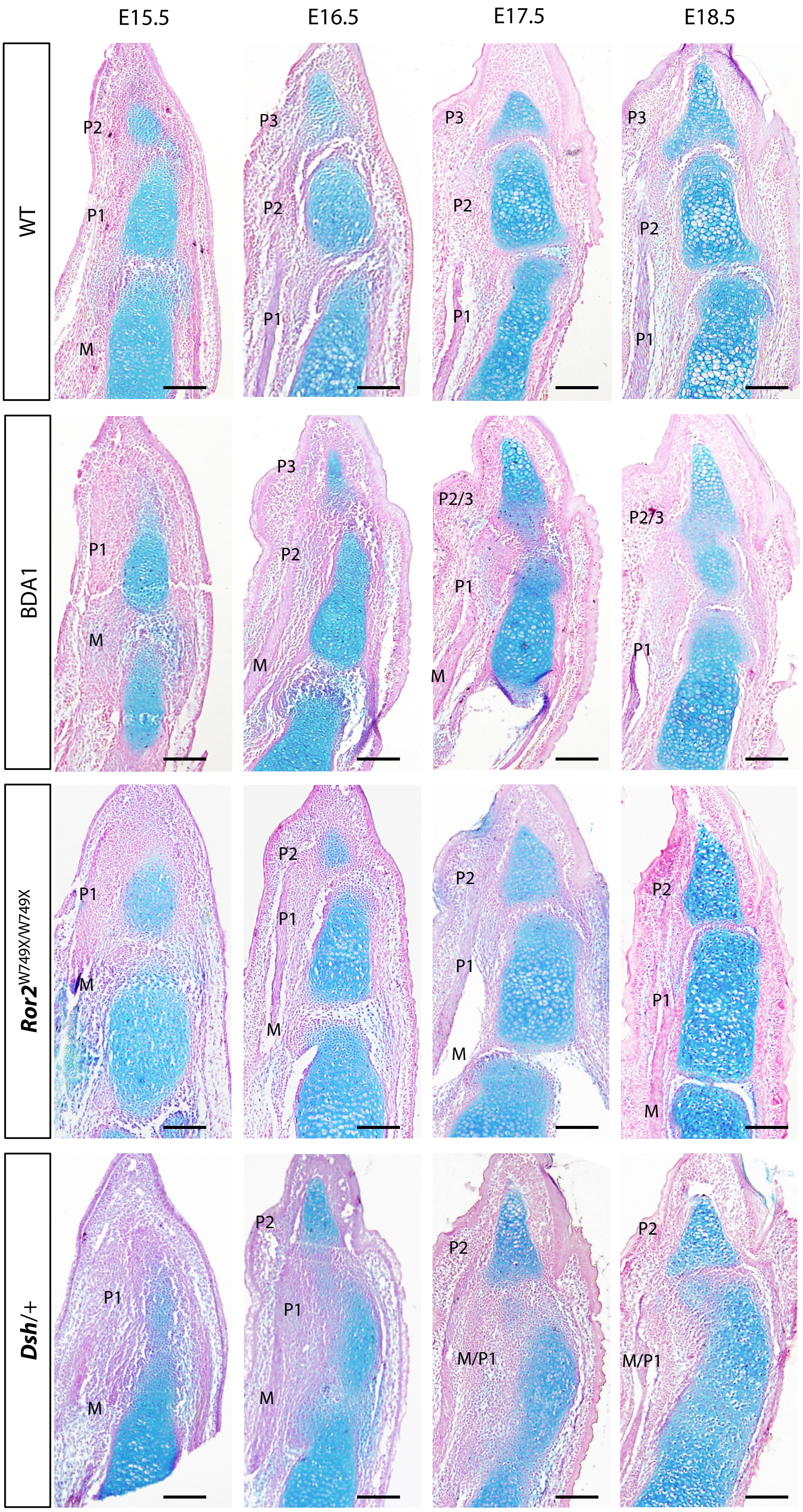

Supplementary Figure S5. **Histological comparison among various Brachydactyly mutants.** Alcian blue and nuclear fast red histological staining of WT, BDA1, *Ror2*$^{W749X/W749X}$ and *Dsh*/+ Digit V, comparing prenatal joint development. While BDA1, *Ror2*$^{W749X/W749X}$ and *Dsh*/+, had common phenotypes of different shortened or missing anlagen, they specifically had missing P2/3, P3 anlage and M/P1 joint respectively. Scale bar = 200 μm.

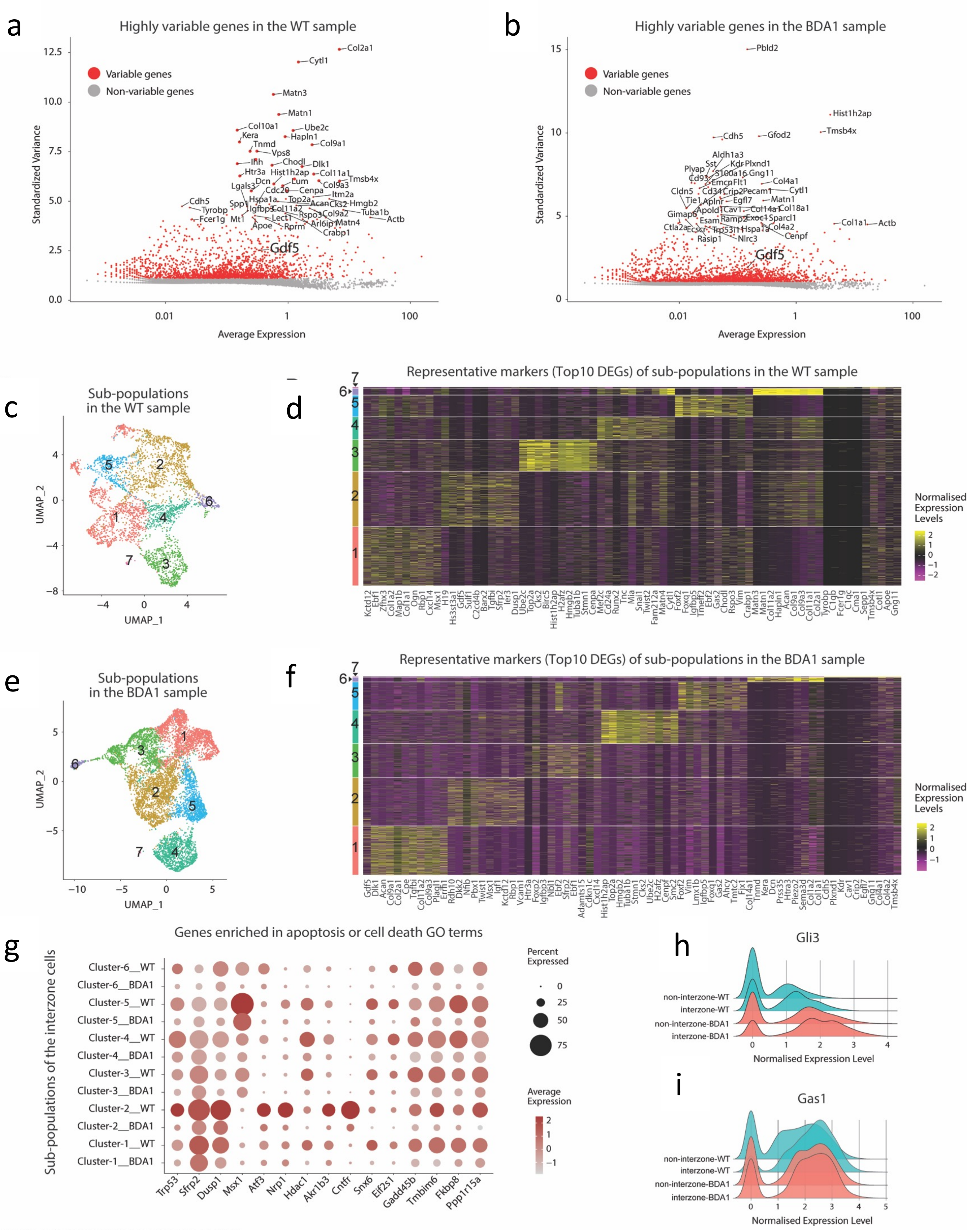

Supplementary Figure S6. **Additional information for the single-cell data. a** Scatter-plot showing the top variable genes in the WT sample. Top 2,000 genes were highlighted red. Top 50 were labelled with gene names. **b** Scatter-plot showing the top variable genes in the BDA1 sample. Top 2,000 genes were highlighted red. Top 50 were labelled with gene names. **c** Dimension reduction of the WT sample by UMAP, showing its first two components and cluster identities from 1 to 7. **d** Heatmap showing the top 10 signature genes of each population in the WT sample. **e** Dimension reduction of the BDA1 sample by UMAP, showing its first two components and cluster identities from 1 to 7. **f** Heatmap showing the top 10 signature genes of each population in the BDA1 sample. **g** A bubble plot showing the expression levels and percentages of the apoptosis or cell death related genes, as revealed by GO analyses in Figure 3J, in the sub-populations of interzone cells across the two genotypes. **h,i** Ridge-plot showing the expression levels of *Gli3* and *Gas1* in the four groups of cells, as labelled on the y-axis.

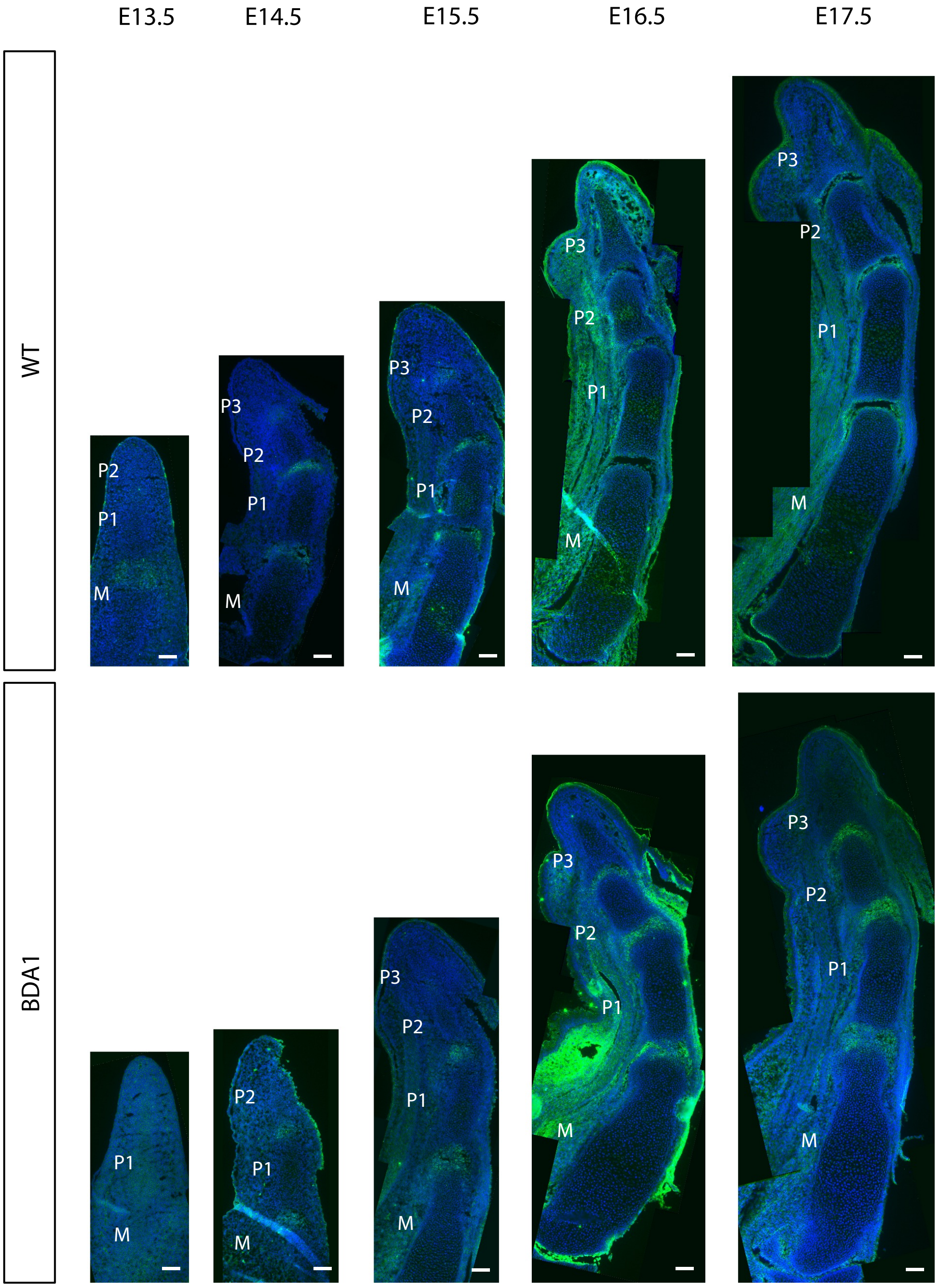

Supplementary Figure S7. **CDON expresses in digit interzones and is up-regulated in BDA1.** Representative immunofluorescence staining of CDON of WT digit III showing CDON expression persisted in the interzone from joint initiation till cavitation. In BDA1, increased CDON expression and broader expression area were observed (n = 3). Scale bar = 100 μm.

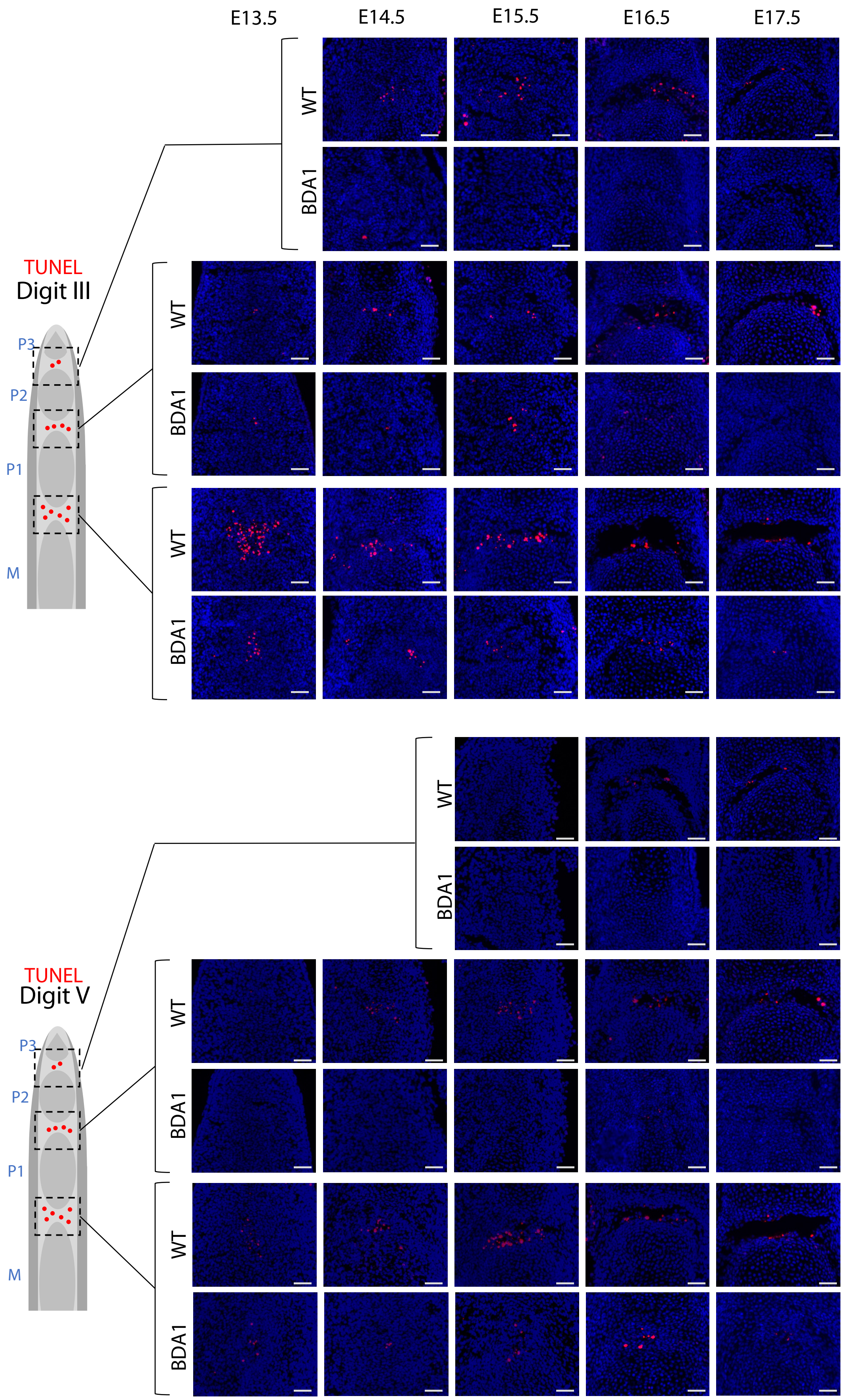

Supplementary Figure S8. **Dynamic cell death patterning of developing digit III and V joints visualized by TUNEL staining.** TUNEL staining of developing digit III and digit V of both WT and BDA1, highlighting cell death trends were specific in joint formation as a programmed stage dependent event. Cell death was reduced in BDA1 across all phalangeal joints from proximal to distal (n = 3). Scale bar = 50 μm.

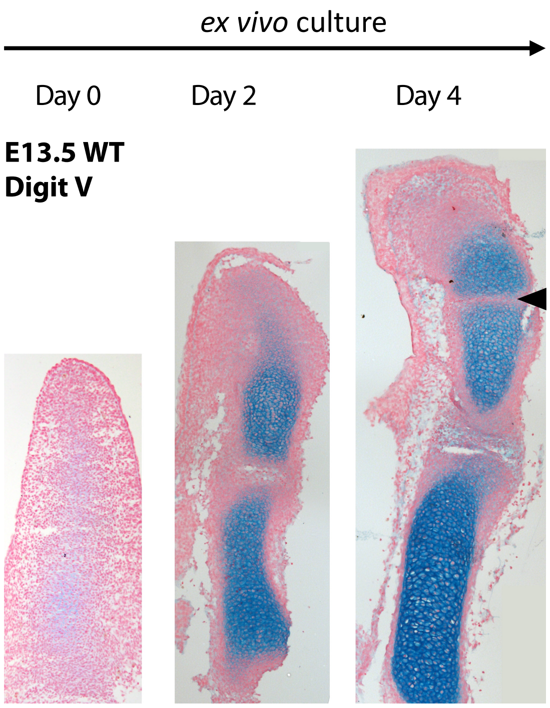

Supplementary figure S9. **Interzone initiation and progression demonstrated by *ex vivo* culture system.** Alcian blue and nuclear fast red histological staining of E13.5 cultured WT digit V, demonstrating anlage and joint development, with the ability for P1/P2 joint initiation and progression (arrow) (n = 5). Scale bar = 100 μm.

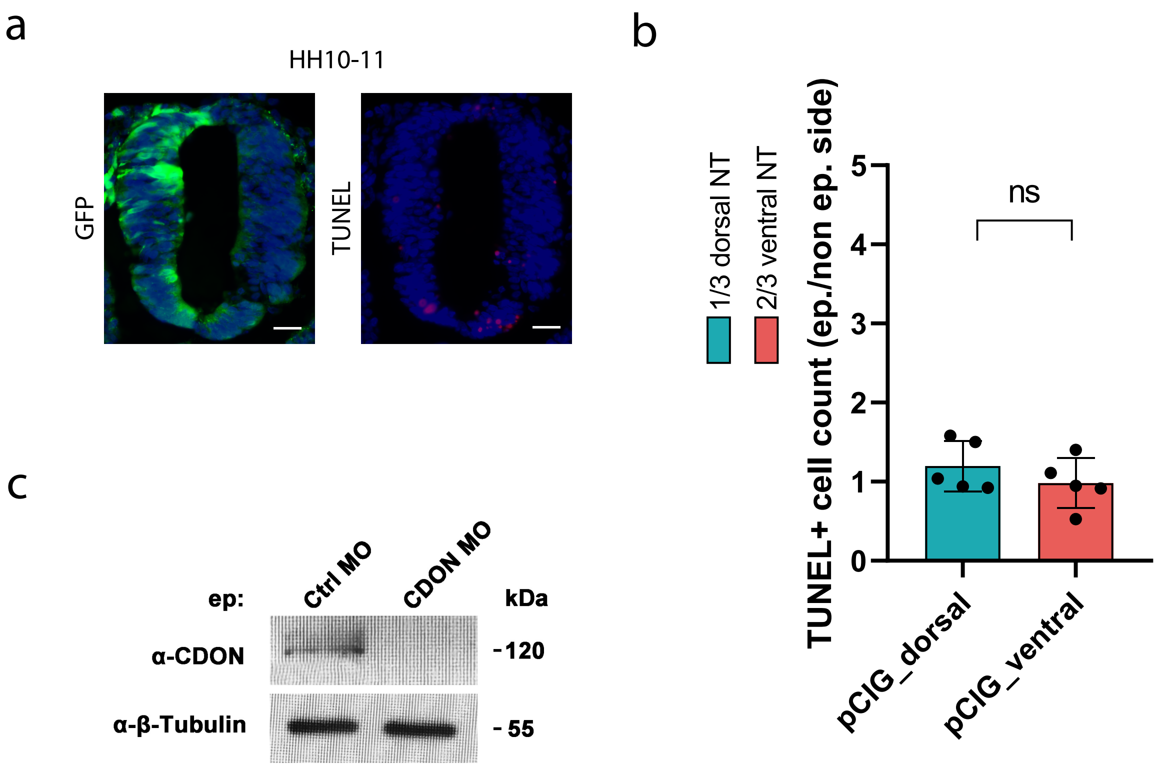

Supplementary figure S10. **Characterization of chick neural tube as a model for studying apoptosis. a** Representative immunofluorescence staining of GFP and TUNEL in chick neural tube at stage HH10-11 that were electroporated with GFP expressing plasmid (pCIG) **b** Bar chart showing TUNEL+ cells in dorsal and ventral chick neural tube. Each data point represents an independent biological replicate, bar height indicates the mean and error bars indicate standard deviations. P-values are calculated with a two-sided student's t-test. The levels of apoptosis were not significantly altered upon electroporation of pCIG. Scale bar = 50 µm. **c** Western blot showing suppression of CDON expression upon CDON MO treatment. β-Tubulin was used as a reference gene. Source data are provided as a Source Data file.

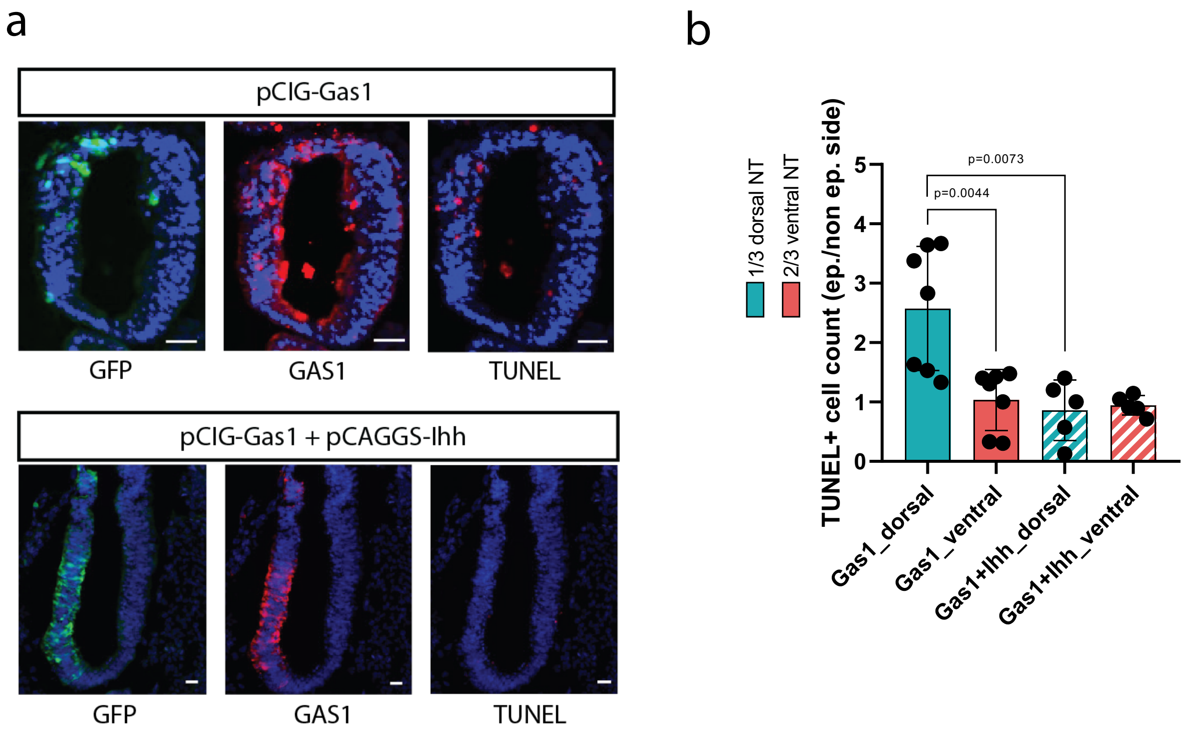

Supplementary figure S11. **Gas1 overexpression in chick neural tube. a** GFP tagged Gas1-expression plasmid with or without *Ihh*-expression plasmids were electroporated to chick neural tubes. Scale bar = 50 µm. **b** Bar chart showing fold change of TUNEL+ cells counts of chick neural tubes electroporated with Gas1- and/or Ihh- expression plasmids. Each data point represents an independent biological replicate, bar height indicates the mean and error bars indicate standard deviations. P-values are calculated with a two-sided student's t-test. Source data are provided as a Source Data file.

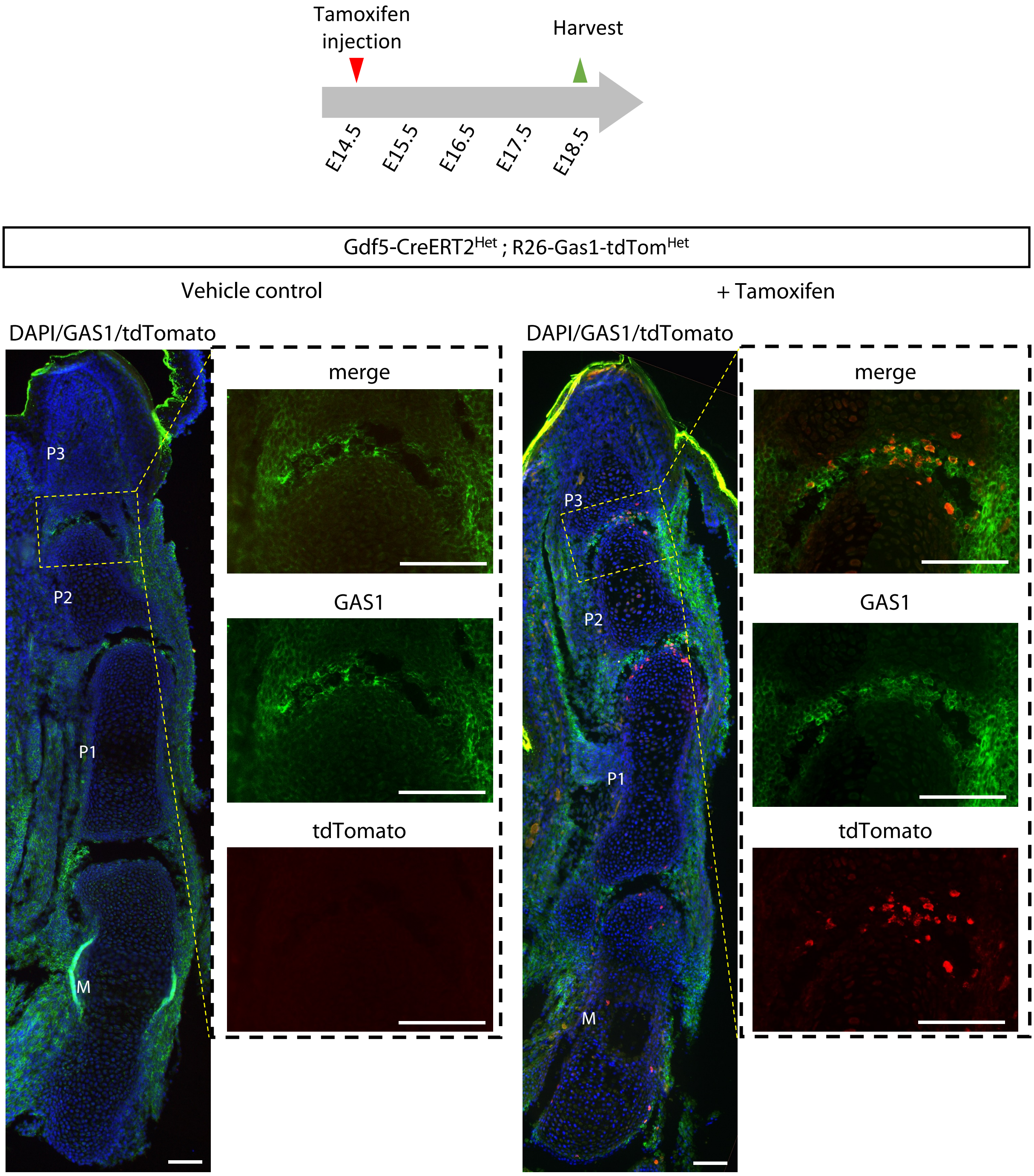

Supplementary figure S12. **Gas1 overexpression in WT digit V.** Representative immunofluorescence staining of GAS1 and tdTomato staining of Gdf5-Cre^{ERT2}; ROSA26$^{Gas1\text{-}Tdtaomto}$ (R26-Gas1-tdTom^{Het}) mouse digit V showing overexpression of GAS1 in Gdf5 lineage cells in digit joints. Scale bar = 100 µm.
