# Supplementary material for: Suppression of apoptosis impairs phalangeal joint formation in the pathogenesis of brachydactyly type A1
Source: Nat Commun. 2024 Mar 12;15:2229. doi: 10.1038/s41467-024-45053-0 (PMC10933404; doi:10.1038/s41467-024-45053-0)
Supplement: Supplementary file 2 — Description of Additional Supplementary Files [file 41467_2024_45053_MOESM2_ESM.pdf]

Description of Additional Supplementary Files.

Supplementary Data 1. List of primers

Supplementary Data 2. Signature genes of the 6 clusters in the interzone.

Supplementary Data 3. Differential expressed genes between interzone and non-interzone, and between WT and mutant.
