# Supplementary material for: Suppression of apoptosis impairs phalangeal joint formation in the pathogenesis of brachydactyly type A1
Source: Nat Commun. 2024 Mar 12;15:2229. doi: 10.1038/s41467-024-45053-0 (PMC10933404; doi:10.1038/s41467-024-45053-0)
Supplement: Supplementary file 4 — Reporting Summary [file 41467_2024_45053_MOESM4_ESM.pdf]

nature portfolio

Corresponding author(s): Danny Chan
Wilson Cheuk Wing Chan

Last updated by author(s): Nov 19, 2023

Reporting Summary

Nature Portfolio wishes to improve the reproducibility of the work that we publish. This form provides structure for consistency and transparency in reporting. For further information on Nature Portfolio policies, see our Editorial Policies and the Editorial Policy Checklist.

Statistics

For all statistical analyses, confirm that the following items are present in the figure legend, table legend, main text, or Methods section.

n/a	Confirmed	
☐	☒	The exact sample size (n) for each experimental group/condition, given as a discrete number and unit of measurement
☐	☒	A statement on whether measurements were taken from distinct samples or whether the same sample was measured repeatedly
☐	☒	The statistical test(s) used AND whether they are one- or two-sided<br>*Only common tests should be described solely by name; describe more complex techniques in the Methods section.*
☒	☐	A description of all covariates tested
☐	☒	A description of any assumptions or corrections, such as tests of normality and adjustment for multiple comparisons
☐	☒	A full description of the statistical parameters including central tendency (e.g. means) or other basic estimates (e.g. regression coefficient) AND variation (e.g. standard deviation) or associated estimates of uncertainty (e.g. confidence intervals)
☐	☒	For null hypothesis testing, the test statistic (e.g. F, t, r) with confidence intervals, effect sizes, degrees of freedom and P value noted<br>*Give P values as exact values whenever suitable.*
☒	☐	For Bayesian analysis, information on the choice of priors and Markov chain Monte Carlo settings
☒	☐	For hierarchical and complex designs, identification of the appropriate level for tests and full reporting of outcomes
☒	☐	Estimates of effect sizes (e.g. Cohen's d, Pearson's r), indicating how they were calculated

Our web collection on statistics for biologists contains articles on many of the points above.

Software and code

Policy information about availability of computer code

Data collection	Fluorescent images were acquired using Olympus BX51 and BX53 fluorescence microscope with SPOT-camera and Hamamatsu digital camera C11440 respectively. Higher-resolution fluorescent images were acquired using Zeiss LSM 880 with Ariyscan 2 inverted confocal microscope. Confocal microscopy images were processed by the ZEN software. For images with multiple staining, different fluorescent channels were merged as indicated in the figure. Histological images were acquired with a Zeiss Axioplan 2 microscope with SPOT-camera and an Olympus BX51 microscope with Promicam USB 3.0 Digital Camera.
Data analysis	Protein sequences from Uniprot were aligned and visualized using Pfaat 2.0.130 and protein interactions were visualized using PyMOL 2.4.0, Single cell transcriptomics raw sequencing data was processed with CellRanger 3.1.0 and transcriptomics data was analyzed using the Seurat package, version 3. Image data, including brightness, contract and color, were adjusted and images were exported using Adobe Photoshop CS. Neural tube signal intensity was quantified by ImageJ. Statistical analyses were performed using GraphPad Prism 8.4.3.

For manuscripts utilizing custom algorithms or software that are central to the research but not yet described in published literature, software must be made available to editors and reviewers. We strongly encourage code deposition in a community repository (e.g. GitHub). See the Nature Portfolio guidelines for submitting code & software for further information.

Data

Policy information about availability of data

All manuscripts must include a data availability statement. This statement should provide the following information, where applicable:

- Accession codes, unique identifiers, or web links for publicly available datasets
- A description of any restrictions on data availability
- For clinical datasets or third party data, please ensure that the statement adheres to our policy

Source data are provided with this paper. The raw data of the scRNA-seq used in this study was deposited on NCBI GEO database (accession number: GSE183253; reviewers' token: kxkhgmiidruhfgn, [https://www.ncbi.nlm.nih.gov/geo/query/acc.cgi?acc=GSE183253]). The processed scRNAseq data is also available on an interactive web interface at: https://www.sbms.hku.hk/dclab/BDA1.

Field-specific reporting

Please select the one below that is the best fit for your research. If you are not sure, read the appropriate sections before making your selection.

☒ Life sciences ☐ Behavioural & social sciences ☐ Ecological, evolutionary & environmental sciences

For a reference copy of the document with all sections, see nature.com/documents/nr-reporting-summary-flat.pdf

Life sciences study design

All studies must disclose on these points even when the disclosure is negative.

Sample size	No statistical method was used to predetermine sample size. The number of animals were determined to ensure the biological replicates for each genotype / mutation studied and were calculated based on Mendelian ratio.
Data exclusions	No data were excluded from the analysis if not explicitly stated.
Replication	Expect sample sizes were indicated in the graphs. For fluorescence images, three staining were performed independently and the representative images were shown.
Randomization	Randomization was not applicable for this study because the phenotype of the animal reported is based on the underlying gene mutation.
Blinding	Not relevant to the study. The phenotype reported is according to the specific mutations. All samples were harvested and processed equally.

Reporting for specific materials, systems and methods

We require information from authors about some types of materials, experimental systems and methods used in many studies. Here, indicate whether each material, system or method listed is relevant to your study. If you are not sure if a list item applies to your research, read the appropriate section before selecting a response.

Materials & experimental systems

n/a	Involved in the study	
☐	☒	Antibodies
☒	☐	Eukaryotic cell lines
☒	☐	Palaeontology and archaeology
☐	☒	Animals and other organisms
☒	☐	Human research participants
☒	☐	Clinical data
☒	☐	Dual use research of concern

Methods

n/a	Involved in the study	
☒	☐	ChIP-seq
☒	☐	Flow cytometry
☒	☐	MRI-based neuroimaging

Antibodies

Antibodies used

Mouse anti-NKX2.2, 74.5A5, Developmental Studies Hybridoma Bank
Mouse anti-PAX6, Pax6, Developmental Studies Hybridoma Bank
Goat anti-CDON, AF2429, R&D Systems
Goat anti-GAS1, AF2644, R&D Systems
Sheep anti-EGFP, 4745-1051, AbD Serotec
Rabbit anti-IHH, NB110-57122, Novus Biologicals
Rabbit anti-HHIP, 12316-R, Bioss
Goat anti-GFP, ab6673, Abcam
Guinea pig anti-COLXXII, gift from Dr. Manuel Koch

Alexa Fluor 488 donkey anti-sheep, A11015, Thermo Fisher
Alexa Fluor 594 goat anti-mouse, A11005, Thermo Fisher
Alexa Fluor 488 rabbit anti-goat, ab150141, Abcam
Alexa Fluor 594 donkey anti-goat, ab150132, Abcam
Alexa Fluor 488 donkey anti-guinea pig, 706-545-148, Jackson Immunoresearch
Alexa Fluor 594 donkey anti-guinea pig, 706-585-148, Jackson Immunoresearch
NothernLights NL637 donkey anti-goat, NL002, R&D Systems

Validation

All commercially available antibodies are validated by their respective manufacturer. Details of the primary antibodies are as follows:

Mouse anti-NKX2.2, 74.5A5, Developmental Studies Hybridoma Bank: Manufacturer's validation: dshb.biology.uiowa.edu/74-5A5, relevant citations: Briscoe J, Cell, 1997, and >20 others

Mouse anti-PAX6, Pax6, Developmental Studies Hybridoma Bank: Manufacturer's validation: dshb.biology.uiowa.edu/PAX6, relevant citation: Briscoe J, Cell, 1997, and >60 others

Goat anti-CDON, AF2429, R&D Systems: Manufacturer's validation: resources.rndsystems.com/pdfs/datasheets/af2429.pdf?v=20211005&_ga=2.185115561.1045387463.1633489669-1345291052.1559793166, relevant citation: Fabre PJ, Shimogori T, Charron F, J. Neurosci, 2010.

Goat anti-GAS1, AF2644, R&D Systems: Manufacturer's validation: resources.rndsystems.com/pdfs/datasheets/af2644.pdf?v=20211005&_ga=2.108659458.1045387463.1633489669-1345291052.1559793166, relevant citation: van Roeyen C et al., Kidney Int, 2012.

Sheep anti-EGFP, 4745-1051, AbD Serotec: Manufacturer's validation: images.bio-rad-antibodies.com/datasheets/datasheet-4745-1051.pdf?_ga=2.7034226.1953185279.1633501208-1101005593.1633501208, relevant citation: Won JH et al., Cell Death Dis., 2019, and >20 others.

Rabbit anti-IHH, NB110-57122, Novus Biologicals: Manufacturer's validation: www.novusbio.com/PDFs/NB110-57122.pdf, relevant citation: Valverde LF et al., Tumour Biol., 2016.

Rabbit anti-HHIP, 12316-R, Bioss: Manufacturer's validation: www.biossantibodies.com/datasheets/bs-12316R.

Goat anti-GFP, ab6673, Abcam: Manufacturer's validation: www.abcam.com/gfp-antibody-ab6673.html, relevant citation: Maruno T et al., Elife, 2021 and >300 others.

Guinea pig anti-COLXXII, gift from Dr. Manuel Koch: relevant citation: Feng C et al., Stem Cell Reports, 2019.

Animals and other organisms

Policy information about studies involving animals; ARRIVE guidelines recommended for reporting animal research

Laboratory animals

Mice
IhhE95K, mixed ICR/C57BL/6 , unspecified sex, at specific time point from embryonic stage to post-natal.
Lgr5-GFP, C57BL/6, unspecified sex, E14.5.
Lgr5-DTR-GFP, C57BL/6, unspecified sex, E14.5.
Cdon-/-, 129/SV, unspecified sex, at specific time point from embryonic stage to post-natal.
Gdf5-CreERT2; R26-Gas1, mixed ICR/C57BL/6, unspecified sex, at E18.5

Chicken
Specific pathogen free eggs, HH10-11 and HH11-12

Wild animals

The study did not involve wild animals.

Field-collected samples

The study did not involve samples collected from the field.

Ethics oversight

All animal works were approved by the Committee on the Use of Live Animals in Teaching and Research of the University of Hong Kong. All procedures performed with approved animal license under the regulation of Department of Health, HKSAR.

Note that full information on the approval of the study protocol must also be provided in the manuscript.
